# Integrated Metabolomics, Network Pharmacology, and Molecular Dynamics Simulations Reveal the Potential Anti-Melanoma Mechanisms of *Inonotus hispidus*

**DOI:** 10.3390/cimb48080814

**Published:** 2026-08-12

**Authors:** Hui Liu, Jihao Liu, Zuohao Ma, Hao Li, Xinli Liu, Deqiang Zhu

**Affiliations:** School of Bioengineering, Qilu University of Technology, Shandong Academy of Sciences, Jinan 250353, China

**Keywords:** Sang Huang, melanoma, targeted metabolomics, network pharmacology, molecular dynamics simulation

## Abstract

Melanoma is a highly aggressive malignancy characterized by pronounced metastatic potential, marked heterogeneity, and complex therapeutic resistance. Although immunotherapy and targeted therapy have improved the prognosis of some patients, their long-term clinical application remains constrained by limited therapeutic responses, drug resistance, and adverse effects. In this study, widely targeted metabolomics, network pharmacology, molecular docking, and molecular dynamics simulations were integrated to systematically investigate the potential mechanisms underlying the anti-melanoma effects of *Inonotus hispidus* SH-18. Fruiting bodies of Sang Huang at the Juvenile, Growth, and Maturation stages were analyzed using UPLC-MS/MS, identifying 1575 metabolites across 13 chemical classes. PCA explained 67.91% of the total variance (PC1, 43.54%; PC2, 24.37%), while the OPLS-DA models yielded Q^2^ values of 0.964, 0.966, and 0.981 for IH-G vs. IH-J, IH-M vs. IH-G, and IH-M vs. IH-J, respectively. A total of 1099 differentially accumulated metabolites were identified, including 304 (140 up-accumulated and 164 down-accumulated), 311 (195 up-accumulated and 116 down-accumulated), and 484 (278 up-accumulated and 206 down-accumulated) in the three respective comparisons. Based on the experimentally detected metabolites, network pharmacology and bioinformatics analyses suggested that the active constituents of Sang Huang may synergistically suppress melanoma cell proliferation, survival, immune evasion, angiogenesis, invasion, and metastasis by regulating multiple pathways and signaling axes, including the TP53-p21 axis. Molecular docking showed that the candidate active compounds exhibited binding potential with core targets, among which ursolic acid displayed favorable binding capacity toward all core targets. Molecular dynamics simulations further supported the stability of these interactions. Notably, ursolic acid was most abundant in samples from the Juvenile stage, suggesting that this stage may be preferred for ursolic acid enrichment and subsequent activity evaluation. These findings suggest the potential therapeutic value of Sang Huang in melanoma treatment.

## 1. Introduction

Melanoma is a malignancy arising from the malignant transformation of melanocytes and can occur in the skin, mucosa, and uvea, with cutaneous melanoma being the most common form [1]. Although melanoma accounts for a relatively small proportion of all skin cancers compared with most non-melanoma skin cancers, it is highly malignant, prone to local invasion and distant metastasis, and represents one of the major causes of skin cancer-related mortality [2,3]. According to GLOBOCAN 2022, approximately 331,700 new melanoma cases and 58,700 melanoma-related deaths were reported worldwide, indicating that melanoma continues to impose a substantial disease burden [4]. The initiation and progression of melanoma are associated with genetic susceptibility, ultraviolet radiation exposure, alterations in the immune microenvironment, and aberrant activation of multiple oncogenic signaling pathways [5,6]. Previous studies have shown that the MAPK signaling pathway is one of the most frequently dysregulated pathways in melanoma, and alterations in key molecules such as BRAF, NRAS, NF1, and KIT can lead to sustained MAPK pathway activation, thereby promoting tumor cell proliferation and survival [7,8]. In addition, PI3K-Akt and JAK-STAT signaling, p53-related apoptotic regulation, MYC-mediated transcriptional regulation, and tumor immune-inflammatory networks have also been closely implicated in melanoma progression, therapeutic resistance, and recurrence [8,9].

In recent years, immune checkpoint inhibitors and BRAF/MEK-targeted therapies have substantially reshaped the therapeutic landscape of advanced melanoma [10,11]. However, primary or acquired resistance still occurs in some patients, and long-term application is limited by treatment-related adverse events [12,13]. Therefore, the screening of bioactive compounds with multi-target synergistic regulatory potential from natural products and traditional medicinal fungi has become an important direction for adjuvant intervention and mechanistic research in melanoma [14,15].

Emerging evidence indicates that β-glucans derived from basidiomycete fungi, such as schizophyllan- and lentinan-related polysaccharides, may participate in tumor immunomodulation by enhancing innate and adaptive immune responses [16,17]. Fungal polysaccharides and protein-bound polysaccharides have also been reported to possess the potential to regulate inflammation-, apoptosis-, and angiogenesis-related processes and may be involved in tumor microenvironment modulation by affecting signals such as vascular endothelial growth factor (VEGF) [17,18]. Moreover, triterpenoids and polyphenols derived from medicinal fungi such as *Ganoderma lucidum* exhibit tyrosinase-inhibitory, antioxidant, and antiproliferative activities, suggesting that fungal secondary metabolites warrant further investigation in pigment regulation and tumor intervention [19].

Sang Huang is a medicinal fungus with a long history of use in traditional East Asian medicine; it has traditionally been classified within the genus *Phellinus*, whereas related medicinal taxa have increasingly been referred to as *Sang Huangporus* in recent years. Modern studies have shown that Sang Huang and related *Phellinus*/*Sang Huangporus* taxa contain diverse chemical constituents, including polysaccharides, flavonoids, triterpenoids, steroids, and phenolic acids, and exhibit antitumor, immunomodulatory, antioxidant, anti-inflammatory, and metabolic regulatory activities [19,20,21]. Because melanoma progression involves multiple biological processes, including proliferation, apoptosis, inflammation, immune escape, and signal transduction, the multi-component and multi-target characteristics of Sang Huang are highly compatible with the research framework of network pharmacology [22].

However, the role of Sang Huang in melanoma intervention has rarely been reported. In this study, widely targeted metabolomics and network pharmacology were integrated to prospectively predict and analyze the potential mechanisms by which Sang Huang may improve melanoma, with the aim of providing a reference and basis for further investigation.

## 2. Materials and Methods

### 2.1. Sample

*Inonotus hispidus* SH-18 (GDMCC No. 62276) (Figure S1) was used as the experimental material in this study, and its ITS sequence was registered under accession number OM438243; the taxonomic and medicinal research foundations of Sang Huang and related *Inonotus*/*Phellinus*/*Sang Huangporus* taxa have been previously reported [23]. This strain was originally isolated from wild *Inonotus hispidus* collected from ancient mulberry trees along the old course of the Yellow River in Dezhou, China, Longitude: 115°45′18″ E to 116°16′05″ E, Latitude: 36°52′38″ N to 37°10′07″ N, and was used in the present study. The substrate used for artificial cultivation was prepared on a dry-weight basis with 80% aged mulberry branch sawdust, 15% wheat bran, and 10% corn flour, and the moisture content was adjusted to 60%. The mycelia were first inoculated into potato dextrose (PD) medium and then collected after shaking cultivation at 25–28 °C and 150 rpm for 8 days. Subsequently, the obtained mycelia were inoculated into polypropylene cultivation bags containing the substrate to promote primordium formation. The cultivation bags were then incubated in darkness at 25 °C and 60–70% relative humidity, and samples were collected after 1 month of cultivation. Fresh Sang Huang fruiting bodies at the Juvenile, Growth, and Maturation stages were harvested from the greenhouse, respectively (Table S1). After collection, the fungal materials were washed with PBS/RNase-free water and cut along the central vertical axis into blocks of 1–2 cm in diameter. The samples were then evenly aliquoted into 15–50 mL sterile centrifuge tubes, snap-frozen in liquid nitrogen for 15 min, and temporarily stored at −80 °C; low-temperature storage is a commonly adopted strategy for maintaining metabolite stability during metabolomic sample pretreatment. When required, sample transportation was conducted on dry ice to minimize metabolite degradation during transit [24].

Biological samples were processed by vacuum freeze-drying to minimize metabolite degradation and improve the consistency of subsequent extraction. After being freeze-dried in a freeze dryer (Scientz-100F, Ningbo Scientz Biotechnology Co., Ltd. Ningbo, China), the samples were ground into a fine powder using a grinder (MM 400, Retsch, Retsch GmbH, Haan, Germany) at 30 Hz for 1.5 min. Subsequently, 50 mg of sample powder was accurately weighed using an electronic balance (MS105DM, Mettler-Toledo, Greifensee, Switzerland) and mixed with 1200 μL of internal standard extraction solution consisting of 70% aqueous methanol pre-cooled to −20 °C. When the sample mass was less than 50 mg, the extraction solution was added at a ratio of 1200 μL per 50 mg of sample. After mixing, the samples were extracted by vortexing for 30 s every 30 min, for a total of six cycles. The samples were then centrifuged at 12,000 rpm for 3 min, and the supernatants were collected and filtered through a 0.22 μm microporous membrane. The filtrates were transferred into injection vials for subsequent UPLC-MS/MS analysis (Tables S2 and S3) [25].

Based on a local metabolite database, qualitative and quantitative mass spectrometric analyses of metabolites in the samples were performed. The characteristic ions of each metabolite were selected using a triple quadrupole mass spectrometer, and their signal intensities were recorded as counts per second (CPS) by the detector. Raw mass spectrometry data files were processed using MultiQuant software 3.0.3 for chromatographic peak integration and correction. The peak area of each chromatographic peak was used to represent the relative abundance of the corresponding metabolite, and all integrated peak area data were exported and saved. To compare the abundance differences of each detected metabolite among different samples, chromatographic peaks corresponding to each metabolite were corrected across samples based on retention time and peak shape information, thereby ensuring the accuracy of qualitative and quantitative analyses [26].

### 2.2. Chromatographic and Mass Spectrometric Conditions

The sample extracts were analyzed using a UPLC-ESI-MS/MS system, consisting of an ultra-performance liquid chromatography system (UPLC, ExionLC™ AD, SCIEX, Framingham, MA, USA) coupled with a tandem mass spectrometry system. The UPLC conditions were as follows: separation was performed on an Agilent SB-C18 column (1.8 µm, 2.1 mm × 100 mm); mobile phase A consisted of ultrapure water containing 0.1% formic acid, and mobile phase B consisted of acetonitrile containing 0.1% formic acid. The gradient elution program was set as follows: the initial conditions were 95% A and 5% B; the gradient was linearly changed to 5% A and 95% B within 9 min and maintained for 1 min; it was then returned to 95% A and 5% B within 1.1 min and maintained for 2.9 min. The flow rate was set at 0.35 mL/min, the column temperature was maintained at 40 °C, and the injection volume was 2 μL. The chromatographic effluent was alternately introduced into an electrospray ionization-triple quadrupole-linear ion trap (ESI-QTRAP) mass spectrometry system for detection, a mode suitable for high-throughput qualitative analysis and relative quantification of metabolites [27].

The ESI source parameters were set as follows: the source temperature was maintained at 500 °C, and the ion spray voltage (IS) was set at 5500 V in positive ion mode and −4500 V in negative ion mode. Ion source gas I (GSI), ion source gas II (GSII), and curtain gas (CUR) were set at 50, 60, and 25 psi, respectively; the collision-activated dissociation (CAD) parameter was set to High. Data acquisition on the triple quadrupole was performed in multiple reaction monitoring (MRM) mode, with nitrogen used as the collision gas and set to Medium. For each MRM transition, the declustering potential (DP) and collision energy (CE) were individually optimized. A specific set of MRM transitions was monitored according to the metabolites eluted within different time windows, thereby improving detection specificity and quantitative stability [27,28].

### 2.3. Quality Control for Widely Targeted Metabolomic Analysis

Quality control (QC) samples were prepared by pooling aliquots of sample extracts and were used to evaluate the reproducibility of samples processed under the same conditions. During instrumental analysis, one QC sample was generally inserted after every 10 analytical samples to monitor the reproducibility of the analytical process. The reproducibility of metabolite extraction and detection, namely technical reproducibility, was assessed by overlaying the total ion chromatograms (TICs) of different QC samples obtained from mass spectrometric analysis (Figure S2A,B). High instrumental stability provided an important basis for data reproducibility and reliability. The coefficient of variation (CV), defined as the ratio of the standard deviation to the mean of the raw data, was used to reflect the degree of data dispersion. The empirical cumulative distribution function (ECDF) was used to evaluate the frequency of metabolites with CV values below a given reference threshold; a higher proportion of metabolites with low CV values in QC samples indicated greater experimental data stability. In the QC samples, more than 85% of metabolites had CV values below 0.5, indicating that the experimental data were relatively stable; moreover, more than 75% of metabolites had CV values below 0.3, indicating that the experimental data were highly stable (Figure S3) [29].

### 2.4. Collection of Chemical Constituents of Sang Huang and Target Prediction

Based on the detected metabolic compounds, the TCMSP database (https://www.tcmsp-e.com/) was searched to screen metabolites and obtain melanoma-related compounds and their corresponding targets. Subsequently, the STITCH database (https://stitch-db.org/) was queried using SMILES strings as search inputs to obtain target information related to compound–protein interactions. Meanwhile, the SwissTargetPrediction database (https://www.swisstargetprediction.ch/) was used to predict the potential targets of small molecules, thereby complementing the results retrieved from TCMSP and STITCH. Finally, the targets obtained from the three databases were integrated, and invalid or duplicate targets were removed. The curated targets were standardized using the UniProt database (https://www.uniprot.org/), and non-human genes were excluded to obtain standardized gene symbols [30].

### 2.5. Acquisition of Melanoma-Related Targets

Disease-related targets were obtained by searching the GeneCards database (https://www.genecards.org/) and the OMIM database (https://omim.org/) using the keyword “melanoma”. All targets retrieved from the two databases were integrated into a spreadsheet, duplicate entries were removed, and the remaining targets were standardized using the UniProt database to obtain disease target information.

### 2.6. Prediction of Overlapping Targets Between Sang Huang and Melanoma

The identified Sang Huang-related targets were mapped to melanoma-related disease targets, and overlapping genes were obtained through Venn diagram analysis. Subsequently, a “drug–compound–target” network was constructed using Cytoscape 3.8.2 to visualize the multinode relationships between compounds and targets.

### 2.7. Protein–Protein Interaction Network Construction and Core Target Identification

To further investigate the protein–protein interactions involved in the potential therapeutic effects of Sang Huang against melanoma, the common drug–disease targets were uploaded to the STRING database (https://string-db.org/) to construct a protein–protein interaction (PPI) network. The organism was set to *H. sapiens*, the minimum required interaction score was set to 0.7, and all other parameters were kept at their default settings. The results were saved in TSV format, imported into Cytoscape 3.8.2, and analyzed using NetworkAnalyzer via Cytoscape → Tools → NetworkAnalyzer → Network Analysis → Analyze Network. Node size and color were used to represent degree values, whereas edge thickness was used to represent the combined score. Core targets were then selected, and the PPI network diagram was constructed [31].

### 2.8. Gene Ontology (GO) and Kyoto Encyclopedia of Genes and Genomes (KEGG) Pathway Enrichment Analyses

The common drug–disease genes were uploaded to the DAVID database (https://davidbioinformatics.nih.gov), with OFFICIAL_GENE_SYMBOL selected as the gene identifier and *Homo sapiens* set as the species. GO functional annotation was performed using DAVID, and the functional characteristics of potential target proteins involved in the effects of Sang Huang against melanoma were analyzed from three aspects: biological process (BP), cellular component (CC), and molecular function (MF). To clarify the signaling pathways associated with Sang Huang-related targets against melanoma, KEGG pathway enrichment analysis was further performed. The top 10 GO terms in the BP, CC, and MF categories, together with 17 melanoma-related KEGG pathways with *p* < 0.01, were selected as the major enriched functional processes and signaling pathways to predict the potential mechanisms by which Sang Huang may exert therapeutic effects against melanoma [32].

### 2.9. Molecular Docking Analysis

Molecular docking between the active constituents of Sang Huang and key targets was performed using AutoDock Vina (version 1.1.2; The Scripps Research Institute, Olson Laboratory, La Jolla, CA, USA) to evaluate their interaction potential [33]. The detailed procedure was as follows: (1) compound structures in SDF format were downloaded from the PubChem database and imported into ChemBio3D for energy minimization. Subsequently, the optimized structures were imported into AutoDockTools 1.5.6 for hydrogen addition, charge calculation and assignment, and rotatable bond setting, and were then saved in PDBQT format. (2) Key target protein structures were downloaded from the PDB database (http://www.rcsb.org/), with priority given to structures derived from human proteins, structures in which the original ligands showed high structural similarity to the candidate active constituents, and structures with high resolution. (3) The protein structures were imported into PyMOL 2.3.0 to remove original ligands and water molecules, and were then imported into AutoDockTools 1.5.6 for hydrogen addition, charge calculation and assignment, atom type specification, and saving in PDBQT format. (4) Protein binding sites were predicted using POCASA 1.1; the grid box size was set to 60 × 60 × 60, the grid spacing was set to 0.375 Å, and all other parameters were kept at their default settings. (5) Interaction modes were analyzed using PyMOL 2.3.0. In general, nodes with higher degree values in a network are considered to have greater topological importance [34]. Therefore, proteins with higher degree values in the PPI network may play more important roles in the potential therapeutic effects of Sang Huang against melanoma.

### 2.10. Molecular Dynamics (MD) Simulation Analysis

Molecular dynamics (MD) simulations were performed using GROMACS 2021.5 in this study. The topological parameters of the receptor proteins were generated using the PDB2GMX tool based on the AMBERGS force field (Garcia & Sanbonmatsu, PNAS 99, 2782–2787, 2002). The topology files of small molecules were constructed using Sobtop, with force-field parameters assigned from the GAFF force field, and hydrogen atoms were added using Avogadro. The simulated protein system was embedded in a solvent box containing the TIP3P water model, with the box boundary placed at least 10 Å from the protein boundary, and an appropriate number of Na^+^ or Cl^−^ ions was added to neutralize the system. After system construction, energy minimization was performed to eliminate unfavorable interatomic contacts and prevent instability during the MD simulation. Subsequently, the solute was restrained in the canonical ensemble (NVT), and the system was heated from 0 to 300 K; equilibration was then performed in the isothermal–isobaric ensemble (NPT) at 300 K and 1 bar. A 75 ns MD simulation was then conducted for each complex, and the simulation trajectories were saved for subsequent analysis. Finally, the root mean square deviation (RMSD) and root mean square fluctuation (RMSF) of the systems were analyzed based on the MD simulation results.

## 3. Results

### 3.1. Differentially Accumulated Metabolites (DAMs) in Sang Huang

**Figure 1:**
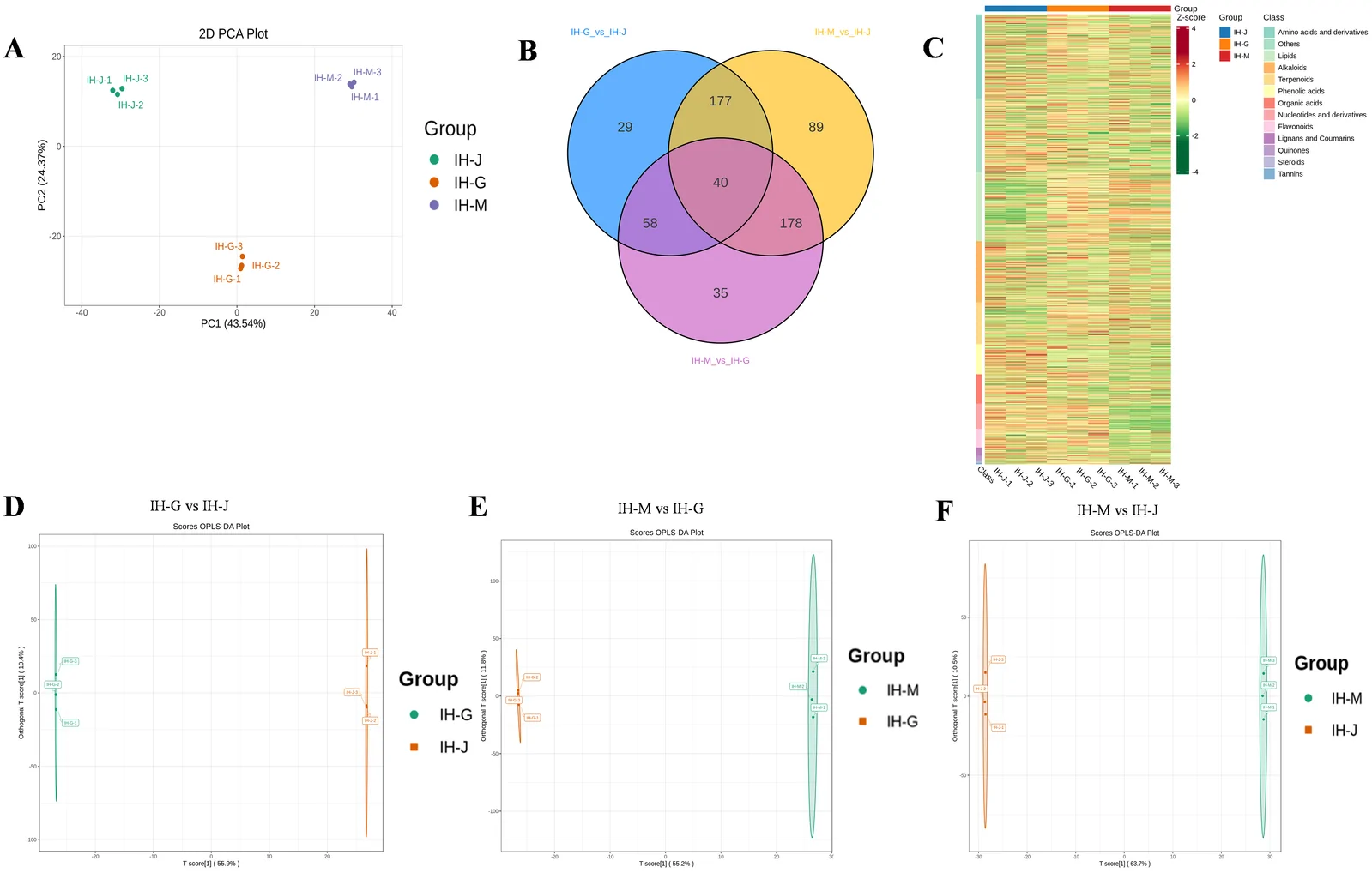
Metabolic profiles of Sang Huang at different developmental stages. (**A**) Principal component analysis (PCA) score plot of metabolites from different samples. (**B**) Venn diagram analysis of differential metabolites among comparison groups. (C) Cluster heatmap of differential metabolites among different samples. (**D**−**F**) Orthogonal partial least squares-discriminant analysis (OPLS−DA) score plots of different comparison groups.

To determine the metabolite composition of Sang Huang at different developmental stages, widely targeted metabolomic profiling was performed using UPLC-MS/MS to characterize differentially accumulated metabolites in Sang Huang at the Juvenile stage (IH-J), Growth stage (IH-G), and Maturation stage (IH-M). After data preprocessing, principal component analysis (PCA) showed that the first two principal components explained 67.91% of the total variance (PC1 = 43.54%, PC2 = 24.37%) (Figure 1A). The PCA score plot showed clear differences in the metabolite profiles of Sang Huang fruiting bodies among different developmental stages, and biological replicates clustered closely together, indicating the reproducibility of the metabolomic data. The metabolomic datasets of individual samples varied across developmental stages, and a total of 1575 metabolites were identified in positive and negative ion modes. These metabolites were classified into 13 chemical categories, including amino acids and their derivatives (294, 19%), phenolic acids (106, 7%), nucleotides and their derivatives (90, 6%), flavonoids (64, 4%), quinones (17, 1%), lignans and coumarins (29, 2%), alkaloids (214, 14%), terpenoids (146, 9%), organic acids (102, 6%), lipids (242, 15%), and others (258, 16%). In addition, OPLS-DA analysis revealed clear separation among IH-J, IH-G, and IH-M samples (Figure 1D–F), highlighting the influence of developmental stage on metabolites in Sang Huang fruiting bodies [35]. A well-fitted and predictive OPLS-DA model was established (Figure S4), and its model fit and validity were evaluated by permutation testing. In this model, 200 random permutation tests were performed; Q2 represents the predictive ability of the model, with Q2 > 0.5 indicating a valid model and Q2 > 0.9 indicating an excellent model, and values closer to 1 indicating greater model stability and reliability. The Q2 values were 0.964 for IH-G vs. IH-J, 0.966 for IH-M vs. IH-G, and 0.981 for IH-M vs. IH-J; all three Q2 values were close to 1, indicating that the model was stable and reliable. Therefore, the model was considered suitable for subsequent screening of differentially accumulated metabolites (DAMs).

### 3.2. Screening and Classification of DAMs

**Figure 2:**
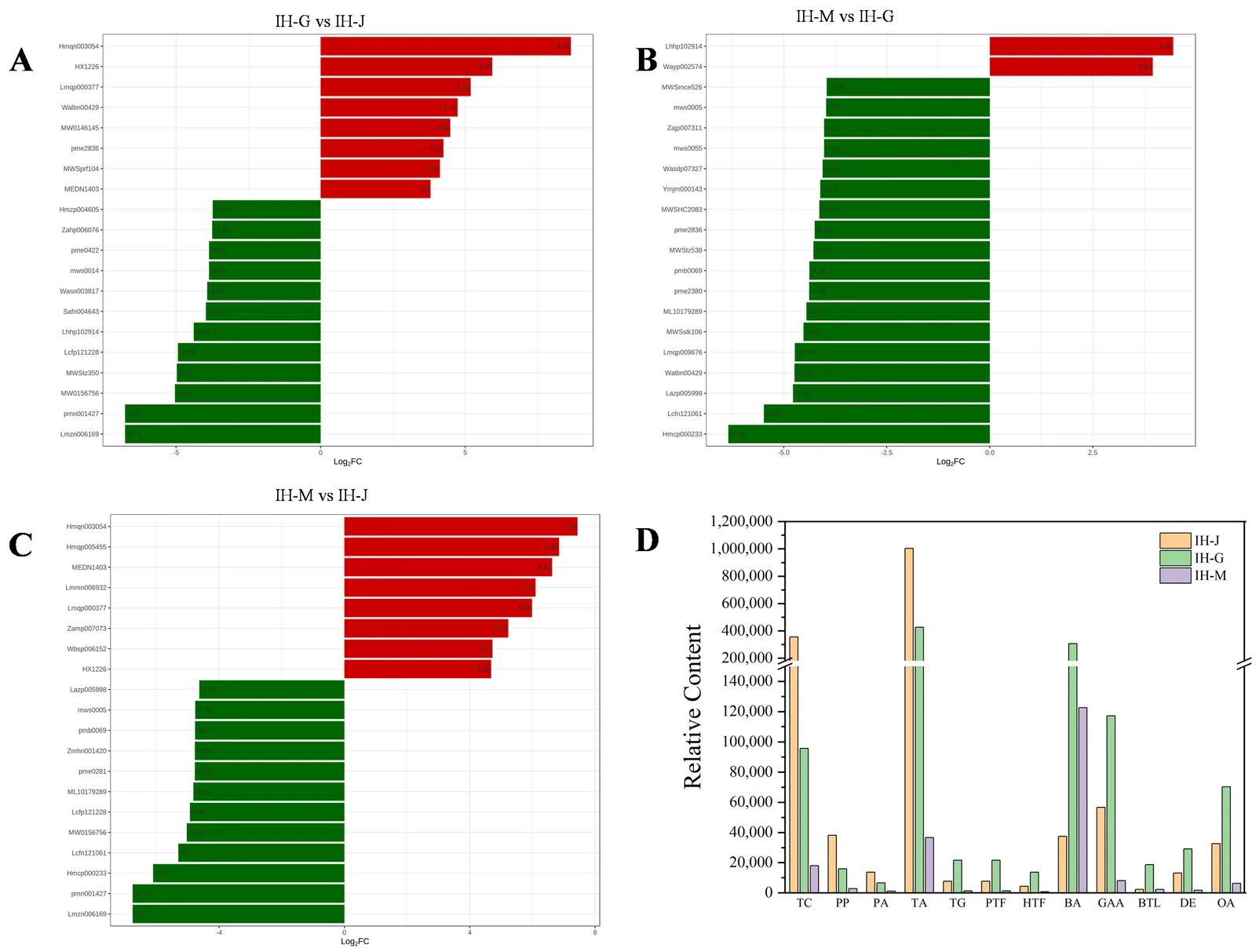
Screening of biomarkers (**A**−**C**) Bar charts of fold changes between different groups. (**D**) Bar charts of biomarkers at different stages. From left to right, the four groups represent phenolic acids, flavonoids, and terpenoids. The *x*-axis represents the abbreviated names of the metabolites: TC for trans-cinnamate, PP for phenylpropanoate, PA for phelligridin A, TA for terephthalate, TG for tangeretin, PTF for 3′,4′,5′,5,7-pentamethoxyflavone, HTF for 3′,4′,5,5′,6,7-hexamethoxyflavone, BA for bonannione A, GAA for ganoderenic acid A, BTL for betulin, DE for 1,6−dihydroxy−4(14)−eudesmene, and OA for orthosphenic acid. The *y*-axis represents the relative content of the metabolites. Red means the metabolite level is up, green means the metabolite level is down.

A total of 1575 metabolites were identified from nine biological samples, and 1099 differentially accumulated metabolites (DAMs) were obtained according to the screening criteria shown in the Venn diagram (Figure 1B). Specifically, 304 DAMs were identified in the IH-G vs. IH-J comparison, including 140 up-accumulated and 164 down-accumulated metabolites (Tables S4–S6). According to the fold-change ranking (Figure 2A–C), among the top 20 metabolites, 9,10,11-trihydroxy-12-octadecenoic acid, 4-hydroxy-β-bulnesene, pyrroline, malic acid-1-O-glucopyranoside, Asp-Ile-Gly, 5-hydroxytryptophol, 4′,6,7-trihydroxyisoflavone, and 10-hydroxy-2-decenoic acid were up-accumulated. In contrast, 3,19-dihydroxyurs-12-en-28-oic acid (pomolic acid), 16,23:16,30-diepoxydammar-24-ene-3,20-diol (jujubogenin), Ser-Gly-Leu, sarmentosin, Ile-Val-Trp, sphaelactone A, ferulate, and isoferulic acid were down-accumulated. In the IH-M vs. IH-G comparison, 311 DAMs were identified, including 195 up-accumulated and 116 down-accumulated metabolites. In the IH-M vs. IH-J comparison, 484 DAMs were identified, including 278 up-accumulated and 206 down-accumulated metabolites. The screening and classification of DAMs reflected pronounced metabolic remodeling in Sang Huang fruiting bodies at different developmental stages, which is consistent with the application of LC-MS/MS-based metabolomics for identifying differential metabolites and characterizing stage-specific metabolic features.

### 3.3. Analysis of DAMs and Biomarker Screening

According to the metabolomic results, 304 differentially accumulated metabolites (DAMs) were identified in the IH-G vs. IH-J comparison, including 140 up-accumulated and 164 down-accumulated metabolites. This result suggested that, during the growth of Sang Huang fruiting bodies, substantial metabolite consumption and biosynthesis may occur simultaneously, reflecting developmental stage-associated metabolic remodeling. In the IH-M vs. IH-G comparison, 311 DAMs were identified, including 195 up-accumulated and 116 down-accumulated metabolites. This result indicated that, when Sang Huang fruiting bodies transitioned from the Growth stage to the Maturation stage, some terminal metabolites tended to accumulate, which is consistent with the use of metabolomics to reveal dynamic metabolic changes across developmental stages.

To identify potential biomarkers in Sang Huang fruiting bodies at different developmental stages, differential metabolite analysis was performed among different comparison groups. Based on the differential grouping of samples, 40 common metabolites were identified among the IH-G vs. IH-J, IH-M vs. IH-G, and IH-M vs. IH-J comparisons (Table S7). Among these metabolites, four phenolic acids, four flavonoids, three alkaloids, five terpenoids, and 16 lipids were identified. In addition, one amino acid and its derivative, one nucleotide and its derivative, and five metabolites classified as others were detected. These common differentially accumulated metabolites (DAMs) covered lipids, terpenoids, flavonoids, phenolic acids, and other small molecules, indicating that the metabolic changes in Sang Huang across different developmental stages were characterized by multiple chemical categories. Representative DAMs included LysoPE 16:1 (2n isomer), LysoPC 17:1, 2-ethynylbenzaldehyde, LysoPE 17:1 (2n isomer), S-adenosyl-L-methionine, ganoderenic acid A, tangeretin, phelligridin A, and 10-hydroxy-2-decenoic acid.

After the common metabolites across the three developmental stages were screened and classified, four flavonoids, four phenolic acids, and four terpenoids were selected as candidate biomarkers. A bar chart was generated based on the relative abundances of these metabolite classes at different developmental stages (Figure 2D). Overall, the relative abundance of phenolic acids gradually decreased from IH-J to IH-M, whereas the relative abundances of flavonoids and terpenoids increased from IH-J to IH-G and then decreased from IH-G to IH-M. These biomarkers reflected the overall dynamic patterns of phenolic acids, flavonoids, and terpenoids, respectively. The identification of candidate biomarkers may contribute to a better understanding of metabolic profile changes in Sang Huang at different developmental stages and may serve as important indicators for monitoring metabolic changes and evaluating the efficacy of Sang Huang fruiting bodies.

### 3.4. Screening of Active Constituents of Sang Huang and Target Prediction

Based on the experimentally detected metabolites, 22 melanoma-related compounds were initially screened using the TCMSP database. After integration and deduplication of results from TCMSP, STITCH, and SwissTargetPrediction, 755 targets were obtained.

### 3.5. Melanoma-Related Targets and Overlapping Targets

**Figure 3:**
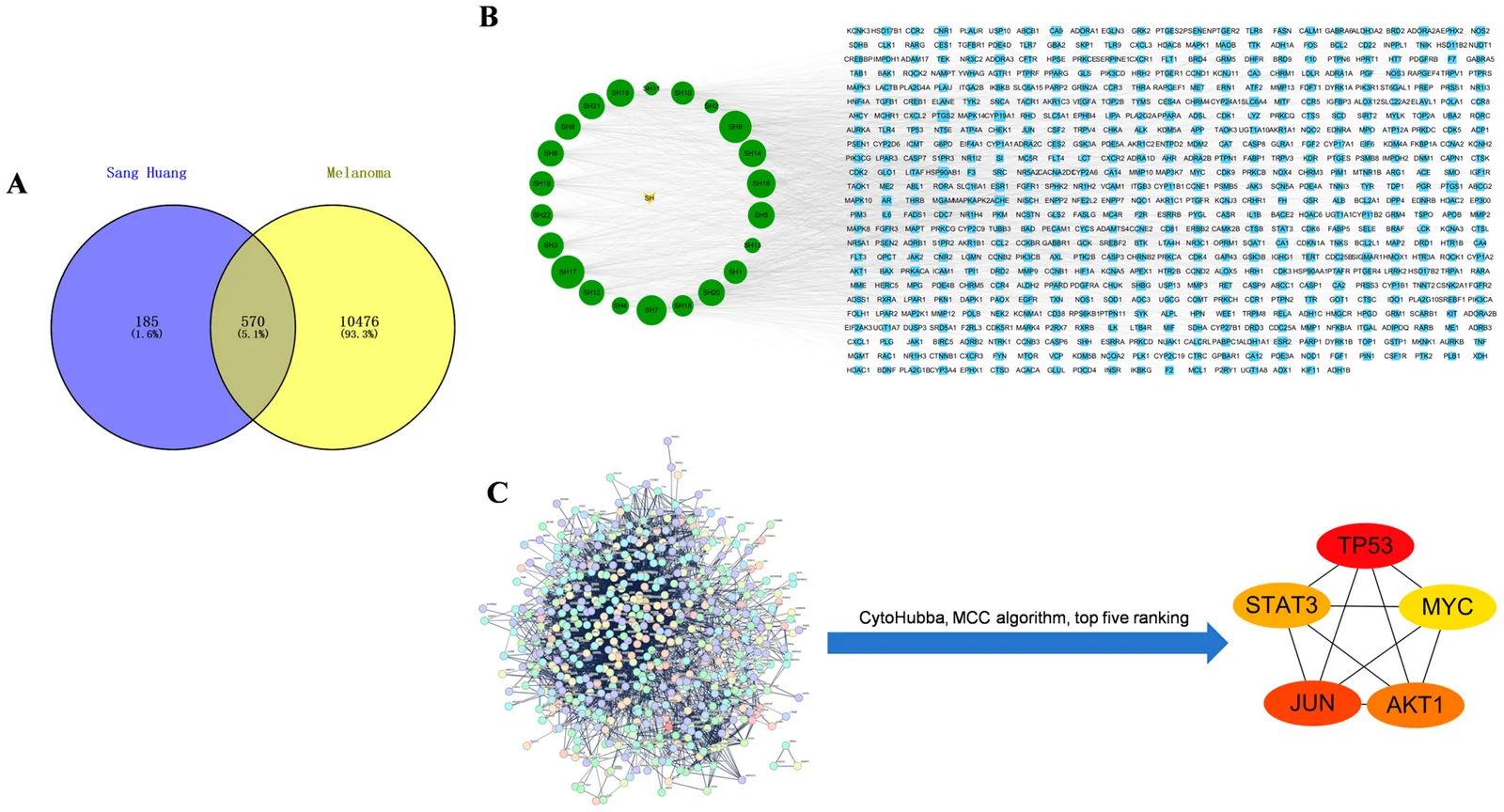
(**A**) Venn Diagram of the Overlapping Targets Between Sang Huang and Melanoma. (**B**) Sang Huang–Compound–Target Network. (Rectangles represent targets, circles represent drug ingredients, and V-shaped nodes represent traditional Chinese medicines). (**C**) Core Target Subnetwork Identified Using the MCC Algorithm.

In the GeneCards database, targets with a relevance score >10 were selected, and 10,360 and 116 melanoma-related targets were obtained from the GeneCards and OMIM databases, respectively. After the union of targets was taken, 10,476 melanoma-related targets were obtained, and the resulting genes were standardized using the UniProt database. As shown in Figure 3A, after the drug-related targets were intersected with melanoma-related target genes, 570 common drug–disease target genes were identified, representing the potential interactive target genes for the treatment of melanoma by the drug.

### 3.6. Construction and Analysis of the Drug–Compound–Target Network

A Sang Huang–compound–target network was constructed based on the compound–target relationships corresponding to the common targets. As shown in Figure 3B, the network consisted of 593 nodes and 1803 edges, including one drug node, 22 compound nodes, and 570 target nodes. Network topology analysis showed that compounds with high degree values mainly included ursolic acid (CAS No. 77-52-1), naringenin (CAS No. 478-01-3), (−)-vestitol (CAS No. 35878-41-2), tamarixetin (CAS No. 603-61-2), and FER (N-feruloyltyramine 4′-glucoside, CAS No. 537-98-4). These compounds may have relatively high connectivity in the compound–target network associated with Sang Huang intervention in melanoma.

### 3.7. Core Target Identification and PPI Network Analysis

After all drug-related targets were intersected with melanoma-associated target genes, 570 common drug–disease target genes were identified, representing potential interactive target genes for melanoma treatment. The 570 common target genes were imported into the STRING database (https://string-db.org/) for protein–protein interaction prediction, with the species set as *Homo sapiens* and the confidence score set to 0.7. The network file was saved in TSV format and imported into Cytoscape 3.8.2 to construct the PPI network. The network contained 543 nodes and 4497 edges.

As shown in Figure 3C, core targets were further screened using the maximal clique centrality (MCC) algorithm in the cytoHubba plugin. The top five ranked targets were TP53 (tumor protein p53), JUN (Jun proto-oncogene), AKT1 (AKT serine/threonine kinase 1), STAT3 (signal transducer and activator of transcription 3), and MYC (MYC proto-oncogene, bHLH transcription factor).

### 3.8. Biological Function Enrichment Analysis

#### 3.8.1. GO Enrichment Analysis

**Figure 4:**
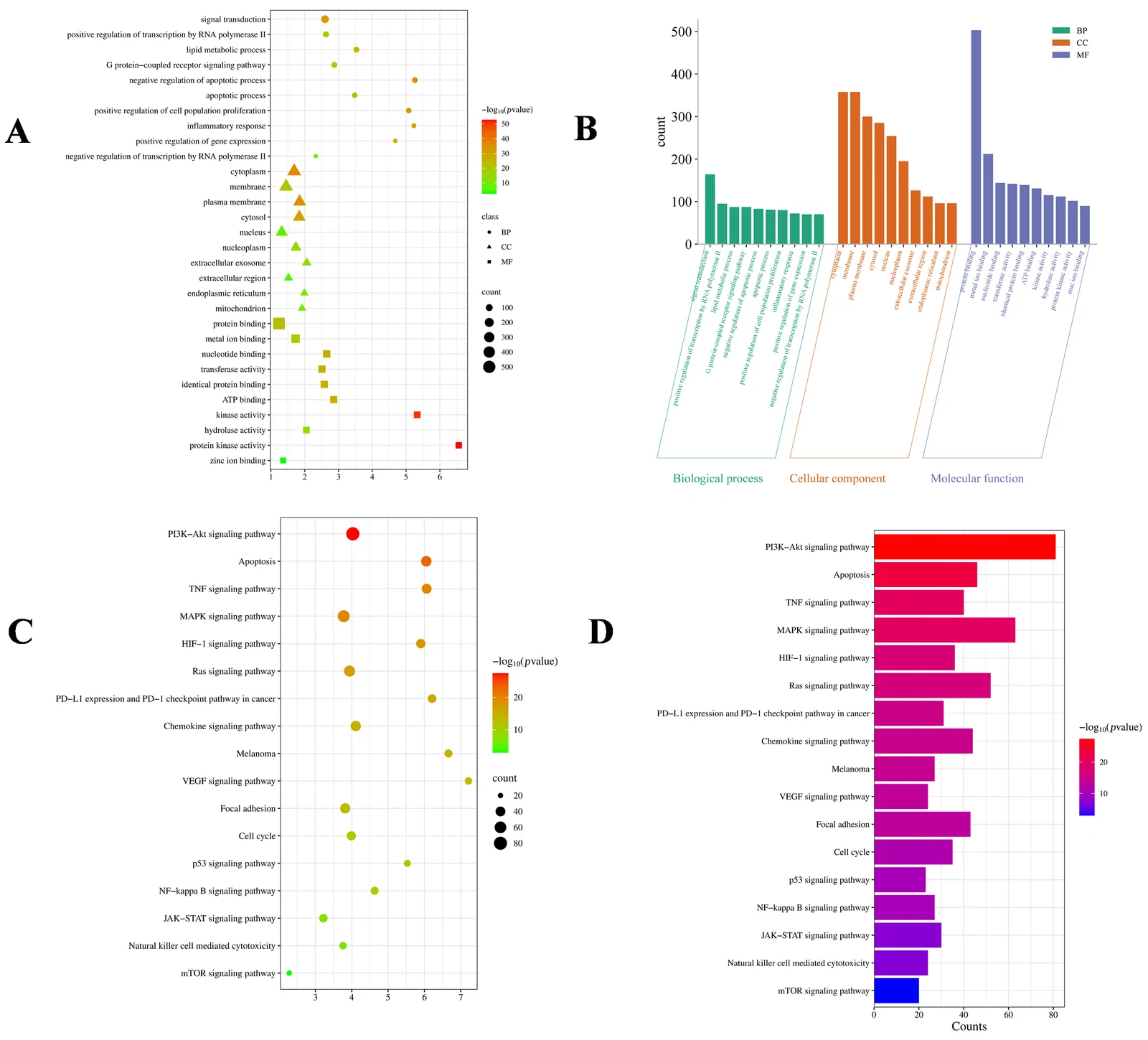
(**A**) Bubble plot of GO enrichment analysis. (**B**) Bar graph of GO enrichment analysis (Note: The larger the degree value, the larger the shape and the darker the color). GO = Gene Ontology. (**C**) Bubble chart for the same analysis (Note: The size of the circle indicates the data of genes enriched in the corresponding pathway, with colorsranging from blue to red indicating progressively smaller *p*-values. (**D**) Bar graph for KEGG pathway enrichment analysis. *p*-value quantifies the probability ofobserving the experimental results (or more extreme outcomes) under the null hypothesis, with lower values (typically <0.05) indicating stronger evidence against the null hypothesis and greater statistical significance. KEGG = Kyoto Encyclopedia of Genes and Genomes.

As shown in Figure 4A,B, GO functional enrichment analysis of the common drug–disease genes was performed using the DAVID database. The 570 common targets showed extensive enrichment at the GO level. A total of 1512 BP terms were enriched, including 847 terms with *p* < 0.01; 171 CC terms were enriched, including 93 terms with *p* < 0.01; and 368 MF terms were enriched, including 187 terms with *p* < 0.01. The BP category was significantly enriched in processes such as positive regulation of MAPK cascade, response to xenobiotic stimulus, negative regulation of apoptotic process, positive regulation of cell population proliferation, signal transduction, and inflammatory response, suggesting that Sang Huang may affect melanoma cell proliferation, apoptosis, inflammatory responses, and signal transduction. The CC category was mainly enriched in structures such as cytoplasm, plasma membrane, cytosol, membrane, and receptor complex, indicating that the targets were distributed in compartments associated with membrane receptors, cytoplasmic signal transduction, and nuclear regulation. The MF category was mainly enriched in functions such as protein kinase activity, kinase activity, protein tyrosine kinase activity, nuclear receptor activity, enzyme binding, and ATP binding, suggesting that protein kinase-related regulation may represent an important molecular basis for Sang Huang intervention in melanoma.

#### 3.8.2. KEGG Pathway Enrichment Analysis

Pathway enrichment analysis was performed using the DAVID database, and 219 pathways associated with the potential therapeutic effects of Sang Huang against melanoma were enriched. Based on a threshold of *p* < 0.01, 186 pathways related to the effects of Sang Huang against melanoma were screened, and pathways potentially associated with melanoma were further selected, as shown in Figure 4C,D. These pathways included the PI3K-Akt signaling pathway, Ras signaling pathway, MAPK signaling pathway, and mTOR signaling pathway, suggesting that proliferation- and survival-related signaling may be involved in Sang Huang-mediated intervention in melanoma. Enrichment of apoptosis, cell cycle, and p53 signaling pathway suggested that cell cycle regulation and apoptosis may represent key processes. Enrichment of PD-L1 expression and PD-1 checkpoint pathway in cancer, JAK-STAT signaling pathway, NF-kappa B signaling pathway, chemokine signaling pathway, and natural killer cell-mediated cytotoxicity suggested that immune-inflammatory regulation and immune escape may also be involved in this process. Enrichment of HIF-1 signaling pathway, VEGF signaling pathway, and focal adhesion indicated that angiogenesis, hypoxic responses, and invasion- and migration-related processes may be affected.

Among the pathways screened above, 17 melanoma-related KEGG pathways were further selected to highlight functional modules more closely associated with melanoma initiation and progression. A bubble plot was then generated based on the *p* values, in which the x-axis represented the number of genes enriched in each pathway, bubble size indicated the number of genes enriched in the corresponding pathway, and color intensity represented statistical significance. This visualization provided an intuitive representation of pathway enrichment and statistical significance, thereby facilitating the overall identification of potential key pathways involved in Sang Huang-mediated intervention in melanoma.

### 3.9. Molecular Docking Results

Based on the preceding analysis, the top five key targets and compounds with high degree values were selected for semi-flexible molecular docking. Binding affinity was evaluated using binding energy, with values below 0 indicating favorable binding between small molecules and target proteins; generally, lower binding energy values suggest a higher likelihood of binding.

**Figure 5:**
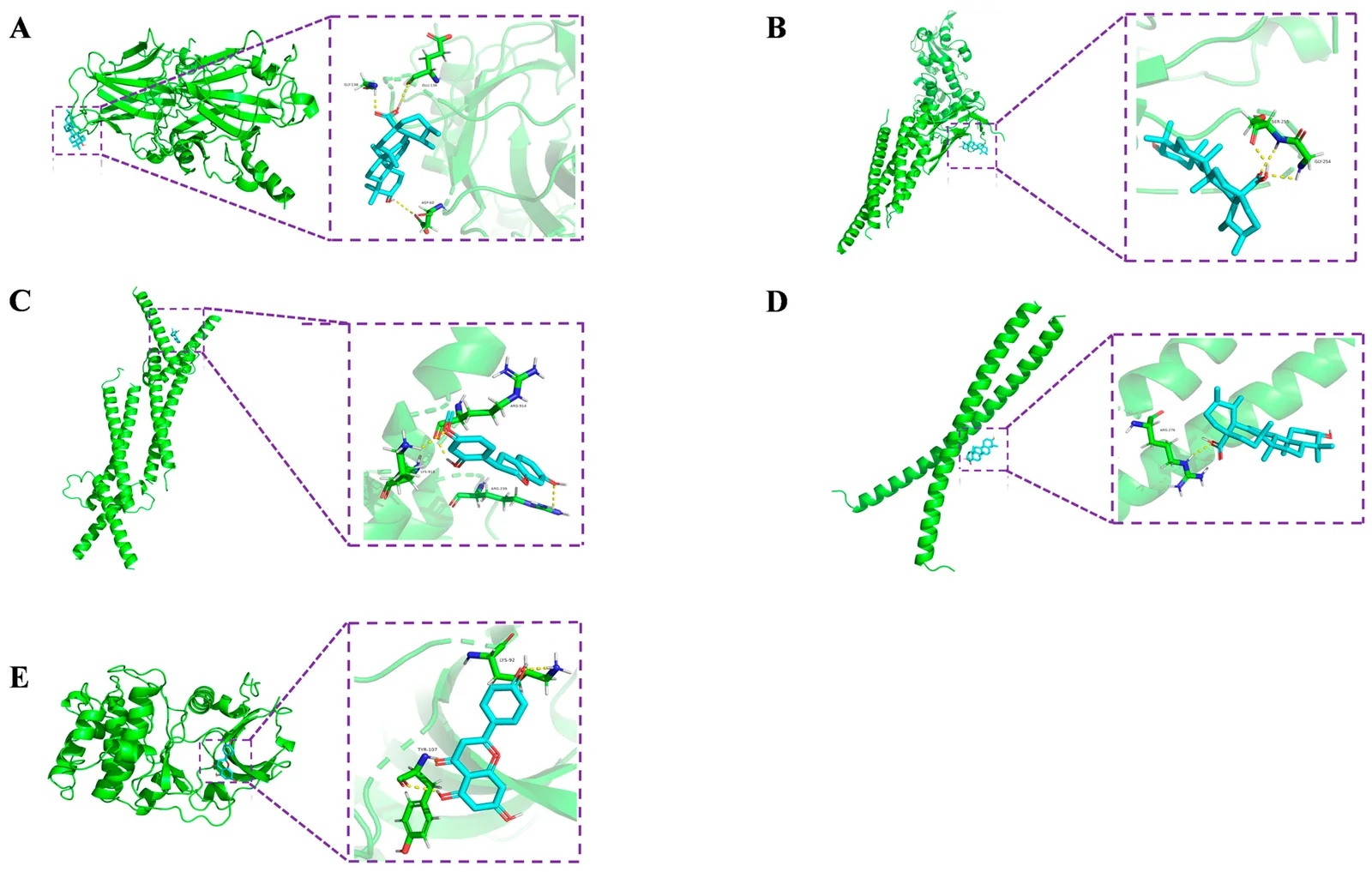
(**A**) ursolic acid and TP53 proteins (**B**) ursolic acid and STAT3 proteins (**C**) ursolic acid and MYC proteins (**D**) ursolic acid and JUN proteins (**E**) ursolic acid and AKT1 proteins.

The docking results showed that the candidate small molecules could enter the active sites of the target proteins, suggesting their potential binding capacity with the core targets. For visualization, the small molecule showing the best docking performance with each protein was selected for graphical presentation (Figure 5). Ursolic acid formed hydrogen bonds with GLU-136 and GLY-138 of TP53, suggesting that it may establish stable interactions within the active pocket of TP53. Ursolic acid formed hydrogen bonds with GLY-254 and SER-255 of STAT3, suggesting that its binding conformation may be maintained through hydrogen-bond interactions. In addition, ursolic acid formed hydrogen bonds with ARG-239, ARG-914, and LYS-918 of MYC, ARG-276 of JUN, and LYS-92 and TYR-107 of AKT1. The docking binding energies between the core small molecules and core target proteins are shown in Table 1.

### 3.10. MD Simulation Results

Based on the molecular docking results, 75 ns molecular dynamics (MD) simulations were performed for the five complex systems, namely ursolic acid–TP53, ursolic acid–STAT3, ursolic acid–MYC, ursolic acid–JUN, and ursolic acid–AKT1, to further investigate their dynamic properties.

Root mean square deviation (RMSD) can be used to evaluate the overall structural deviation of proteins or protein–ligand complexes during simulations and is an important indicator for assessing system stability. RMSD values are commonly expressed in angstroms or nanometers; lower RMSD values indicate smaller deviations among different conformations in the trajectory and greater structural similarity.

Root mean square fluctuation (RMSF) is used to quantify the time-dependent fluctuations of atomic positions during molecular dynamics simulations and can reflect the flexibility or rigidity of local protein structures. Higher RMSF values indicate greater dynamic flexibility in specific regions, such as flexible loops or functional domains, which may be involved in conformational changes or molecular recognition; in contrast, regions with lower fluctuations, such as α-helices or β-sheets, usually correspond to structurally stable functional modules. This analysis provides an important basis for elucidating the relationship between protein dynamic properties and functional relevance.

#### Complex Stability Analysis

Based on the molecular docking results, 75 ns molecular dynamics (MD) simulations were performed on five complexes, namely ursolic acid–TP53, ursolic acid–STAT3, ursolic acid–MYC, ursolic acid–JUN, and ursolic acid–AKT1, to further evaluate the dynamic stability of the docked complexes. The evaluation metrics mainly included root mean square deviation (RMSD) and root mean square fluctuation (RMSF), where RMSD reflected overall conformational deviation and RMSF reflected local residue flexibility.

**Figure 6:**
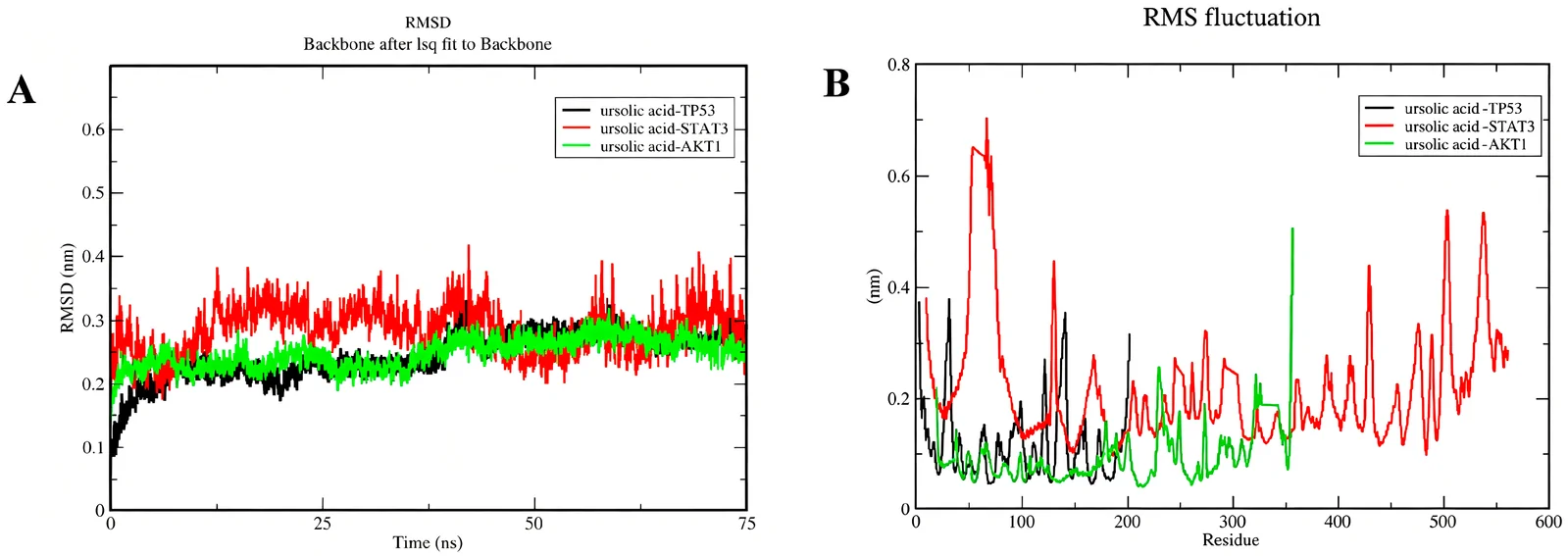
(**A**) RMSD plot based on molecular dynamics simulation. (**B**) RMSF diagram based on molecular dynamics simulation. RMSD = root mean square deviation, RMSF = root mean square fluctuation.

As shown in Figure 6A, the RMSD values of the ursolic acid–TP53, ursolic acid–STAT3, and ursolic acid–AKT1 complexes remained within relatively low ranges throughout the 75 ns simulation, without a sustained increasing trend, suggesting that all three systems exhibited a certain degree of conformational stability. Among them, the ursolic acid–TP53 complex rapidly reached a stable plateau after a short relaxation period, with RMSD values mainly maintained at 0.20–0.27 nm, indicating relatively stable overall structure. The RMSD curve of the ursolic acid–AKT1 complex was relatively smooth and was mainly maintained at 0.22–0.30 nm, indicating that favorable dynamic equilibrium was maintained during the simulation. In contrast, the ursolic acid–STAT3 complex exhibited larger RMSD fluctuations and showed higher transient peaks at later stages, suggesting greater conformational flexibility and relatively lower stability. As shown in Figure 6B, the RMSF results further indicated that most residues in the TP53 and AKT1 complexes exhibited low fluctuations, suggesting relatively stable protein backbones. In AKT1, distinct peaks were observed only in local regions, which may correspond to flexible loop regions or terminal regions. In the STAT3 complex, however, higher RMSF peaks were observed across multiple residue intervals, particularly around residues 50–80 and near residue 500, indicating substantial local flexibility in this system. Taken together, the RMSD and RMSF analyses showed that the ursolic acid–TP53 and ursolic acid–AKT1 complexes exhibited favorable dynamic stability, whereas the ursolic acid–STAT3 complex generally maintained its binding state but showed more pronounced conformational fluctuations and relatively weaker stability. The ursolic acid–MYC and ursolic acid–JUN complexes may have shown instability in their overall conformations because of large intrinsic motions of the protein chains or domains, excessively short protein fragments, or insufficient structural constraints.

### 3.11. Analysis of Bioactive Constituents in Sang Huang

In this study, widely targeted metabolomics was first used to systematically characterize the chemical composition of Sang Huang fruiting bodies at different developmental stages, thereby providing an experimental basis for subsequent network pharmacology-based screening of bioactive constituents and prediction of therapeutic targets. Unlike approaches that solely rely on database-derived compound information, widely targeted metabolomics can reflect the actual metabolic characteristics of Sang Huang fruiting bodies at different developmental stages based on experimentally detected sample data, thereby improving the specificity and reliability of bioactive constituent screening. Sang Huang fruiting bodies at the Juvenile, Growth, and Maturation stages were systematically analyzed using widely targeted metabolomics, and significant differences in metabolic profiles were observed among the different developmental stages. Both PCA and OPLS-DA analyses showed that samples from the three stages could be clearly distinguished, with good clustering of biological replicates, indicating that developmental stage was an important factor affecting metabolite accumulation in Sang Huang and that the obtained data had good stability and interpretability. A total of 1575 metabolites were identified and assigned to 13 chemical classes, among which amino acids and their derivatives, lipids, alkaloids, terpenoids, phenolic acids, and flavonoids accounted for relatively high proportions. These findings suggested that Sang Huang possesses a complex multi-component chemical basis, providing metabolomic evidence for its potential anti-melanoma effects through a “multi-component, multi-target, and multi-pathway” mode of action. Further analysis of differentially accumulated metabolites showed that 1099 differentially accumulated metabolites were screened among the three stages, with 304, 311, and 484 metabolites identified in the IH-G vs. IH-J, IH-M vs. IH-G, and IH-M vs. IH-J comparisons, respectively. These results indicated that Sang Huang fruiting bodies underwent continuous and marked metabolic remodeling during development from the Juvenile stage to the Maturation stage. In terms of dynamic trends, the relative abundance of phenolic acids gradually decreased from IH-J to IH-M, whereas the relative abundances of flavonoids and terpenoids increased from IH-J to IH-G and then decreased from IH-G to IH-M. This pattern suggested that the Growth stage may represent a more active period for the biosynthesis of certain secondary metabolites, whereas the Juvenile stage may retain relatively high levels of early-stage defensive or bioactive constituents.

By integrating the results of network pharmacology, molecular docking, and molecular dynamics simulations, the potential functional significance of key bioactive constituents could be further clarified. Although terpenoids as a chemical class showed a higher accumulation trend at the IH-G stage, ursolic acid, as an individual core compound, exhibited the highest relative abundance at the Juvenile stage, approximately 7.89-fold higher than that at the Growth and Maturation stages. This finding indicated that the overall trend of a chemical class does not necessarily represent the accumulation pattern of key individual compounds. Therefore, the screening of bioactive constituents should comprehensively consider metabolite abundance, compound–target network connectivity, molecular docking affinity, and molecular dynamics stability. As a pentacyclic triterpenoid, ursolic acid showed high connectivity in the compound–target network and favorable binding capacity with core melanoma-related targets, including TP53, AKT1, and STAT3. In particular, the ursolic acid–TP53 and ursolic acid–AKT1 complexes maintained low RMSD and RMSF values during the 75 ns molecular dynamics simulations, suggesting relatively stable conformations. Taken together, the high accumulation of ursolic acid at the Juvenile stage, together with its strong target-binding potential, supports a mechanistic logic linking “high-abundance bioactive constituents, core target binding, and anti-melanoma effects.” These findings suggest that the Juvenile stage of Sang Huang may be a preferred sampling stage for ursolic acid enrichment and subsequent activity validation. However, the anti-melanoma effects of Sang Huang should not be attributed to a single compound alone. The metabolomic results showed that multiple classes of metabolites, including flavonoids, terpenoids, phenolic acids, lipids, and alkaloids, were significantly altered across samples from different stages, suggesting that ursolic acid may be one representative key constituent, whereas other differentially accumulated compounds may participate in synergistic effects through antioxidant and anti-inflammatory activities, apoptosis regulation, and signal transduction modulation. Future studies should integrate absolute quantification using authentic standards, comparative activity evaluation of extracts from different developmental stages, and intervention experiments using individual compounds and compound mixtures to further clarify the contributions of ursolic acid and other differential metabolites to the anti-melanoma effects of Sang Huang.

## 4. Discussion

Metabolomics is an important omics technology for characterizing changes in small-molecule metabolites in cells and tissues under specific physiological or patho-logical conditions, and it has substantial value in revealing developmental stage-dependent and secondary metabolic variations in macrofungi. LC-MS/MS-based metabolomics has been widely applied to the identification of differential metabolites, metabolic pathway analysis, and biomarker screening. Previous studies have applied metabolomics to the compositional characterization of various edible and medicinal fungi and plant resources, including Lentinula edodes, Ganoderma lucidum, other macro-fungi, and medicinal plant samples, thereby contributing to a better understanding of metabolite composition, quality variation, and the chemical basis of bioactivity. There-fore, the integration of widely targeted metabolomics with network pharmacology, molecular docking, and molecular dynamics simulations may help explain the potential anti-melanoma mechanisms of Sang Huang through an analytical chain linking “ex-perimentally detected constituents, potential targets, functional pathways, and struc-tural stability”.

With the continuous iteration of artificial intelligence algorithms and the rapid accumulation of multimodal data, bioinformatics and network pharmacology are evolving from static association analysis toward more comprehensive knowledge mining and predictive modeling [36]. At the algorithmic model level, biomedical language models based on the Transformer architecture, such as BioGPT, have been applied to biomedical text generation, relation extraction, and literature mining, indicating that large-scale pretrained models may provide new computational tools for mining drug–target–disease relationships. Meanwhile, geometric deep learning has also been used for predicting protein–ligand binding conformations; for example, the EquiBind framework uses equivariant graph neural networks to directly predict the binding pos-es of small molecules with proteins, highlighting the value of structure-informed pre-diction of drug–target interactions [37]. At the multi-omics level, spatial transcriptomics, spatial omics, and single-cell sequencing technologies are improving the resolution of tumor microenvironment research, thereby providing a new technical basis for analyzing immune cell heterogeneity, spatial organization, and multi-target synergistic mechanisms.

The developmental-stage variation observed in this study is consistent with previous metabolomic evidence from medicinal fungi. Qi et al. reported marked differences between Sanghuangporus vaninii mycelia and fruiting bodies and among different harvest stages [35], while Zan et al. demonstrated that Inonotus hispidus contains chemically diverse constituents with predicted antitumor relevance [23]. These studies support the biological plausibility of developmental remodeling in Sang Huang. However, differences in species, strain, cultivation substrate, harvest criteria, and analytical platform preclude direct comparison of individual metabolite abundances. The present data also show that the accumulation pattern of a broad chemical class does not necessarily represent that of a specific compound. Therefore, the comparatively high abundance of ursolic acid at the Juvenile stage supports this stage as a candidate for ursolic acid-oriented sampling, rather than establishing it as universally optimal for all bioactive constituents. This interpretation should be verified by absolute quantification and stage-matched bioactivity assays. Moreover, although the use of experimentally detected metabolites narrows the candidate pool compared with database-only screening, the downstream prioritization remains computational and requires preclinical validation [38]. It is important to note that this study relies on computational predictions and metabolomic profiling. While rigorous quality controls (QC samples) were applied to ensure data reliability, in vitro and in vivo experiments with standard positive and negative controls are required in future studies to experimentally validate the predicted anti-melanoma effects.

The functional relevance of the prioritized compounds can be interpreted by comparison with experimental studies rather than by network ranking alone. Ursolic acid has been shown to induce mitochondria-dependent apoptosis in M4Beu human melanoma cells through disruption of the Bax/Bcl-2 balance and activation of caspase-3 [39]. Naringenin has also been reported to induce melanoma-cell apoptosis and inhibit angiogenesis in B16F10 and SK-MEL-28 models [40]. These findings are consistent with the apoptosis- and angiogenesis-related functions suggested by the present analysis and with the recognized importance of p53-associated regulation in melanoma [41]. Nevertheless, evidence obtained using isolated compounds cannot demonstrate that Sang Huang extracts achieve comparable effective concentrations or that the detected constituents act synergistically. Network degree should therefore be regarded as a prioritization metric rather than evidence of pharmacological potency.

At the target level, TP53, JUN, AKT1, STAT3, and MYC are better interpreted as interconnected regulatory nodes than as independent endpoints. These proteins collectively link cell-fate control, survival and metabolic adaptation, stress-responsive transcription, and inflammatory signaling. Their convergence is consistent with the established roles of p53-, MAPK-, PI3K-Akt-, and JAK-STAT-related networks in melanoma progression and therapeutic resistance [32,41]. However, topological ranking only reflects a target’s position in the constructed interaction network. It does not determine whether a constituent activates or inhibits that target, whether the predicted interaction occurs in melanoma cells, or whether its biological consequence is favorable in a specific melanoma genotype. Thus, modulation of these core targets by Sang Huang remains a mechanistic hypothesis requiring direct experimental validation.

The GO enrichment findings are most informative when interpreted as functional convergence. Enrichment of proliferation-, apoptosis-, inflammatory-response-, and kinase-related terms indicates that the predicted targets cluster within biological processes already implicated in melanoma development and treatment response [6,8,32]. This correspondence strengthens the biological coherence of the target set, but it does not constitute independent confirmation that Sang Huang regulates each process. Because GO categories share genes and vary substantially in annotation density, the enrichment pattern may partly reflect functional redundancy and database bias. Accordingly, these terms should guide the selection of experimental endpoints, such as apoptosis, cell-cycle progression, inflammatory signaling, and kinase activation, rather than be presented as demonstrated biological effects.

A similar caution applies to the KEGG results. Concurrent enrichment of Ras/MAPK and PI3K-Akt-mTOR signaling suggests possible convergence on melanoma-cell proliferation and survival, whereas p53 and cell-cycle pathways provide a plausible link to cell-fate regulation. Enrichment of JAK-STAT, NF-kappa B, HIF-1/VEGF, and focal-adhesion pathways further raises testable hypotheses concerning immune regulation, angiogenesis, invasion, and migration, all of which are relevant to melanoma biology [8,9,32]. Nevertheless, over-representation analysis does not establish the direction, magnitude, or temporal order of pathway regulation. Consequently, effects such as upstream dual blockade, PD-L1 downregulation, improvement of the immunosuppressive microenvironment, and suppression of metastasis should be described as predictions to be tested rather than as established actions of Sang Huang.

Molecular docking and molecular dynamics simulations provided an additional prioritization layer. The agreement between the relatively favorable docking scores of ursolic acid and the comparatively stable ursolic acid-TP53 and ursolic acid-AKT1 trajectories strengthens the rationale for experimental follow-up. However, AutoDock Vina binding energies are scoring estimates rather than direct evidence of biochemical binding [33], and trajectory stability only indicates that a modeled complex can remain conformationally stable under the selected simulation conditions. The larger fluctuations observed for the MYC and JUN systems may reflect protein construct length, domain motion, or insufficient structural constraints as well as ligand behavior. In addition, because the metabolites were assigned from chromatographic and mass-spectral features without confirmation using authentic standards or NMR spectroscopy, they should be regarded as putatively annotated in accordance with metabolomics reporting principles [42]. Future studies should confirm prioritized compounds by reference standards, determine their absolute concentrations, compare stage-specific extracts in melanoma cells and normal melanocytes, and verify direct target engagement and pathway modulation before in vivo evaluation.

## 5. Conclusions

In this study, widely targeted metabolomics was used to systematically characterize fruiting body samples of Sang Huang at three developmental stages, namely IH-J, IH-G, and IH-M, and a total of 1575 metabolites were identified, providing an experimentally detected constituent basis for subsequent network pharmacology analysis. Among these metabolites, terpenoids, flavonoids, phenolic acids, and other chemical classes with diverse bioactive potential provided a sample-derived material basis for subsequent network pharmacology and molecular simulation analyses. Molecular docking and molecular dynamics simulation results indicated that Sang Huang constituents, such as ursolic acid, showed relatively stable binding potential with TP53 and AKT1, supporting their possible roles in the mechanisms underlying melanoma intervention. The potential mechanism by which Sang Huang acts against melanoma is characterized by multi-component, multi-target, and multi-pathway features. This study suggested that TP53, AKT1, and STAT3 may be key targets, and that signaling pathways such as the PI3K-Akt pathway may play important roles in this process. However, these predictions still require further validation through in vitro and in vivo experiments.

## Figures and Tables

**Table 1:** Docking results of core small molecules with core target proteins.

Target	PDB ID	Binding Energies of Different Compounds with Targets (kcal/mol)				
		(−)-Vestitol	Tamarixetin	FER	Ursolic Acid	Naringenin
TP53	2XWR	−5.7	−6.3	−5.0	−6.9	−6.2
STAT3	6NJS	−7.2	−8.0	−5.7	−8.1	−7.2
MYC	1NKP	−5.8	−6.0	−4.9	−6.4	−5.8
JUN	5T01	−5.6	−6.0	−5.5	−7.1	−6.2
AKT1	6NPZ	−6.5	−7.1	−5.5	−7.8	−7.3

## Data Availability

The original contributions presented in this study are included in the article/Supplementary Materials. Further inquiries can be directed to the corresponding author.

## Supplementary Materials

The following supporting information can be downloaded at: https://www.mdpi.com/article/10.3390/cimb48080814/s1.
