# Supplementary material for: Integrated Metabolomics, Network Pharmacology, and Molecular Dynamics Simulations Reveal the Potential Anti-Melanoma Mechanisms of Inonotus hispidus
Source: Curr Issues Mol Biol. 2026 Aug 12;48(8):814. doi: 10.3390/cimb48080814 (PMC13510311; doi:10.3390/cimb48080814)
Supplement: Supplementary file 1 [file cimb-48-00814-s001.zip › cimb-4474079-supplementary.pdf]

Supplementary material

Table S1 Sample Identification Information

Species	Stage	Sample	Group
Inonotus hispidus	Juvenile	IH-J-1	IH-J
Inonotus hispidus	Juvenile	IH-J-2	IH-J
Inonotus hispidus	Juvenile	IH-J-3	IH-J
Inonotus hispidus	Growth	IH-G-1	IH-G
Inonotus hispidus	Growth	IH-G-2	IH-G
Inonotus hispidus	Growth	IH-G-3	IH-G
Inonotus hispidus	Maturation	IH-M-1	IH-M
Inonotus hispidus	Maturation	IH-M-2	IH-M
Inonotus hispidus	Maturation	IH-M-3	IH-M

Table S2 Reagent

Name	CAS Code	Purity	Brand
Methanol	67-56-1	Chromatographic Pure	Merck
Acetonitrile	75-05-8	Chromatographic Pure	Merck
Formic Acid	64-18-6	Chromatographic Pure	Aladdin

Table S3 Instrument Details

Name	Model	Brand	Origin
Centrifuge	5424R	Eppendorf	Hamburg, Germany
Thermoblock Mixer	MU-G02-0448	Hangzhou Mio Instruments Co., Ltd	Hangzhou, China
Analytical Balance	MS105DM	Mettler-Toledo Instruments Co., Ltd	Zurich, Switzerland
Centrifugal Concentrator	CentriVap	LABCONCO	Missouri Kansas, USA
Vortex Mixer	VORTEX-5	Kyllin-Bell	Haimen, China
Ultrasonic Cleaner	KQ5200E	Kun Shan Ultrasonic Instruments Co., Ltd	Kunshan, China
Pipette	Research plus	Eppendorf	Hamburg,

			Germany
Automated Workstation	Biomek i5	Beckman Coulter	California, USA
Sealer	Mini HES	Monad	Suzhou, China

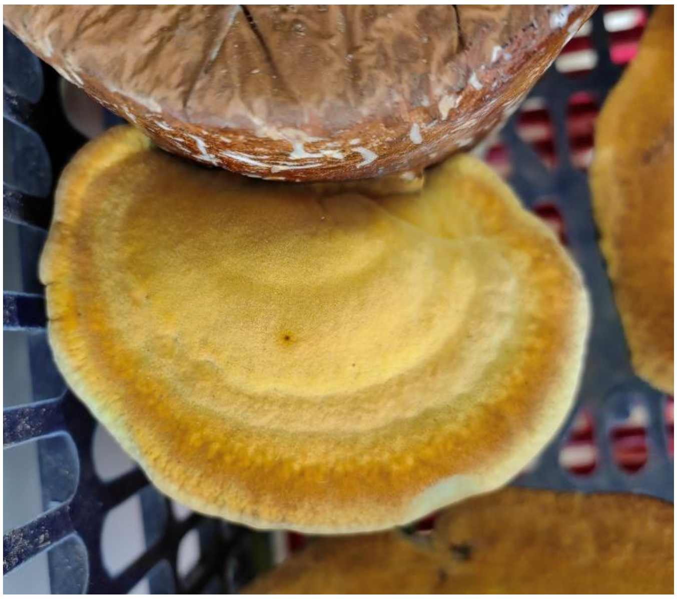

Figure S1 *Inonotus hispidus* SH-18

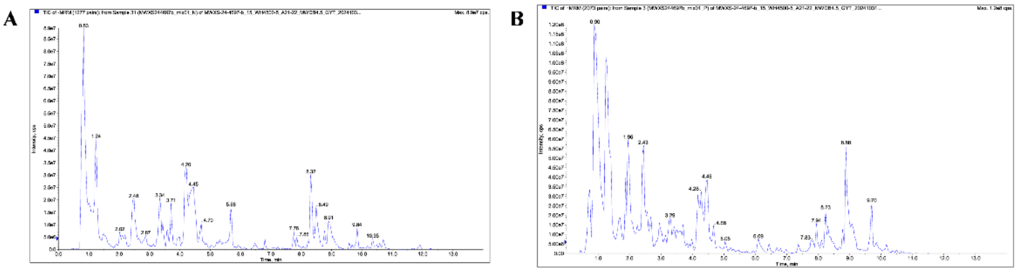

Figure S2(A) QC_MS_TIC-N. (B) QC_MS_TIC-P. (N stands for negative ion mode, P stands for positive ion mode)

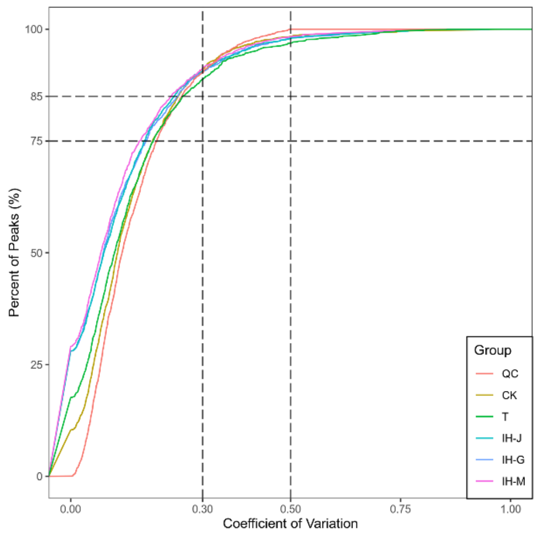

Figure S3: CV Distribution Chart for Each Group of Samples

The x-axis represents the CV values, and the y-axis indicates the proportion of substances with CV values less than the corresponding value out of the total number of substances. Different colors represent different groups of samples.

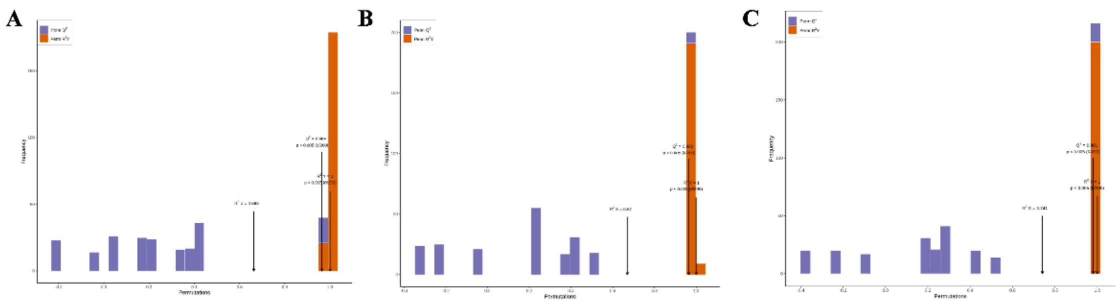

Figure S4： OPLS-DA Validation Plot

（A-C）The x-axis represents the model R^2Y and Q^2 values, and the y-axis indicates the frequency of classification effects observed in 200 random permutation experiments. The orange color represents the R^2Y values of the random grouping models, the purple color represents the Q^2 values of the random grouping models, and the black arrow indicates the original model's R^2X, R^2Y, and Q^2 values.

Table S4 IH-G VS IH-J analysis of differential metabolites results

Compounds	Class I	Type

Biotin	Others	up
6,7-dimethoxy-1-methylisoquinoline	Alkaloids	down
Vasicine	Alkaloids	down
5-Methoxyindoleacetate	Alkaloids	down
Tuliposide D	Others	down
LysoPE 16:1(2n isomer)	Lipids	up
Glycidyl Linoleate	Lipids	up
LysoPC 17:2(2n isomer)	Lipids	up
Harman-3-carboxylic acid	Alkaloids	down
LysoPC 17:1	Lipids	up
Vitetrifolin H	Terpenoids	up
LysoPE 18:2	Lipids	up
LysoPC 17:2	Lipids	up
2-Linoleoylglycerol	Lipids	up
L-Tryptophanamide	Amino acids and derivatives	down
3-amino-2-naphthoic acid	Alkaloids	down
naphthisoxazol A	Alkaloids	down
3-Indoleacrylic acid	Alkaloids	down
2-methyl-1-nitronaphthalene	Others	down
Pyran[3,4-b]indole-2-ketone	Alkaloids	down
(Oxiran-2-yl)methyl octadeca-9,12-dienoate	Lipids	up
Pantothenate	Others	down
LysoPE 18:4	Lipids	up
2-Ethynylbenzaldehyde	Others	down
LysoPE 18:0(2n isomer)	Lipids	up
Peonidin-3-O-arabinoside	Flavonoids	up
Riboflavin	Others	down
LysoPE 17:1(2n isomer)	Lipids	up
Glu-Ile-Val	Amino acids and derivatives	up
Octadeca-2,9,12,15-tetraen-1-ol	Others	up
9R-hydroxy-10E,12Z-octadecadienoic acid	Lipids	up
LysoPE 18:3(2n isomer)	Lipids	up
LysoPE 18:1(2n isomer)	Lipids	up
2,3-Dihydroxypropyl 2-[(octadec-9-enoyl)amino]ethyl hydrogen phosphate	Lipids	up
Dibutylamine	Alkaloids	down
Acetoxy-[6]-gingerol	Phenolic acids	up
Methyl-[8]-gingerol	Phenolic acids	up
Aflatrem	Alkaloids	up
L-isoleucyl-L-aspartyl-L-isoleucine	Amino acids and derivatives	up
NG,NG-Dimethyl-L-arginine	Amino acids and derivatives	up
ethyl-L-arginine	Amino acids and derivatives	up
N,N'-Dimethylarginine;SDMA	Amino acids and derivatives	up
L-Ornithine	Amino acids and derivatives	up

N',N'',N'''-p-Coumaroyl-cinnamoyl-caffeoyl spermidine	Alkaloids	up
4-hydroxy-2-oxo-1,2-dihydroquinoline-3-carboxylic acid	Alkaloids	down
Betaine	Alkaloids	up
His-Glu-Leu	Amino acids and derivatives	down
N(6)-OH-Me-Adenosine	Nucleotides and derivatives	down
4-amino-5-oxo-5-(pentylamino)pentanoic acid	Amino acids and derivatives	down
A-hydroxyquinoline	Alkaloids	down
cyclo-(Gly-Phe)	Amino acids and derivatives	down
3,5-Dihydro-2H-Furo[3,2-C]Quinolin-4-One	Alkaloids	down
3,4-Dihydroxy-L-phenylalanine	Amino acids and derivatives	up
S-Adenosyl-L-methionine	Amino acids and derivatives	down
Homoproline	Amino acids and derivatives	up
L-Cystathionine	Amino acids and derivatives	up
Linolenyl Alcohol	Others	up
Guanosine-5'-monophosphate	Nucleotides and derivatives	up
4,5-Epoxy-14-hydroxy sinomenine N-oxide	Alkaloids	up
Isocytosine	Nucleotides and derivatives	up
Epigoitrin	Others	up
Indole-3-acetate	Alkaloids	down
LysoPE 18:3	Lipids	up
praeroside I	Lignans and Coumarins	up
N-Methyl-Trans-4-Hydroxy-L-Proline	Amino acids and derivatives	down
CDP-choline	Nucleotides and derivatives	down
3-Hydroxy-L-phenylalanine	Amino acids and derivatives	up
10-Formyltetrahydrofolate	Alkaloids	up
5,6-DiHETrE[(±)5,6-dihydroxy-8Z,11Z,14Z-eicosatrienoic acid]	Lipids	up
4-carbamoyl-2-(octadeca-9,12-dienamido)butanoic acid	Lipids	up
21β-Hydroxy-27-nor-C(14a)-homogammacera-14(15)-ene-3,16-dione	Terpenoids	up
4-Hydroxypipecolic acid	Alkaloids	down
Nardosinone	Terpenoids	up
N-Methyl-α-aminoisobutyric acid	Amino acids and derivatives	up
Glu-Ser	Amino acids and derivatives	up
L-Lysine-Butanoic Acid	Amino acids and derivatives	up
γ-Glu-Trp	Amino acids and derivatives	down
Sarmentosin	Alkaloids	down
3-(Pyrazol-1-yl)-L-alanine	Amino acids and derivatives	down
Retronecine	Alkaloids	down
His-Tyr	Amino acids and derivatives	down
7-Hydroxy-4-chromone	Others	down
Isocurcumenol	Terpenoids	up
LysoPE 20:4	Lipids	up
LysoPC 19:1	Lipids	up

3-Hydroxyanthranilate	Alkaloids	down
N-Hexanoyl nornicotine	Alkaloids	up
7-phenylhept-2-en-1-ol	Others	up
9-hydroxy-4,8-dimethylnona-3,7-dienal	Terpenoids	up
N-Caffeoylputrescine	Alkaloids	up
Asp-Ile-Leu	Amino acids and derivatives	up
L-Histidine	Amino acids and derivatives	down
Ala-Ile-Ala	Amino acids and derivatives	up
Nobiletin	Flavonoids	up
2-Oxo-3,4-dihydro-1H-quinoline-3-carboxylic acid	Alkaloids	down
5α,6α,7β,10β-11α,13-dihydro-4(15)-eudesmene-12,6-olide Tsoongiodendroonolide	Terpenoids	up
Brevianamide F	Alkaloids	up
m-Aminophenylacetylene	Alkaloids	down
N-Caffeoylmethylgluconamide	Alkaloids	up
6-[(7-hydroxy-2-methyl-6-oxo-3,4-dihydroisoquinolin-1-yl)methyl]-2,3-dimethoxybenzoic acid	Alkaloids	up
Gly-Val-Ala	Amino acids and derivatives	down
Pro-Gly-Val	Amino acids and derivatives	up
2'-Deoxyinosine 5'-phosphate	Nucleotides and derivatives	down
Vanillylamine	Alkaloids	up
Dihydroisopelletierine	Alkaloids	down
[4]-Shogaol	Phenolic acids	up
12,15-dioxo-selina-4,11-dine	Terpenoids	up
Ganoderenic Acid A	Terpenoids	up
Tangeretin	Flavonoids	up
3',4',5',5,7-Pentamethoxyflavone	Flavonoids	up
Glutinosone	Terpenoids	up
Ganoderic Acid Sigma	Terpenoids	up
Asp-Ile-Gly	Amino acids and derivatives	up
5-Methoxy-1,7-diphenyl-3-heptanone	Others	up
5-Hydroxyquinoline	Alkaloids	up
L-Cyclopentylglycine	Amino acids and derivatives	up
N-(1-Deoxy-1-fructosyl)isoleucine	Amino acids and derivatives	up
Met-Asn	Amino acids and derivatives	down
L-Cystine	Amino acids and derivatives	down
Abscisic acid D-glucopyranosyl ester	Terpenoids	up
Pyrroline	Alkaloids	up
Val-His	Amino acids and derivatives	up
Nicotinic Acid Methyl Ester(Methyl Nicotinate)	Alkaloids	down
(1,3)-1-Methyl-1,2,3,4-tetrahydro-β-carboline-3-carboxylic acid	Alkaloids	up
Ladanetin-6-O-β-D-glucoside	Flavonoids	down
[(1R,2S)-1-(1,3-benzodioxol-5-yl)-2-methyl-3-	Lignans and Coumarins	up

oxobutyl]4-hydroxy-3-methoxybenzoate		
3S,4R,5S,8R,10R-tetrahydroperezinone	Terpenoids	up
2-Hydroxy-2,3-dihydronaphthalene-1,4-dione	Quinones	down
4-Hydroxy-β-bulnesene	Others	up
Caffeoylcholine	Alkaloids	up
5-methyl 1-propyl L-glutamate	Amino acids and derivatives	down
Eremofukinone	Terpenoids	up
Isorhamnetin 3-glucuronide	Flavonoids	up
Ser-Gly-Leu	Amino acids and derivatives	down
His-Asp	Amino acids and derivatives	up
cycloalliin	Amino acids and derivatives	down
5-Acetylsalicylic acid	Phenolic acids	down
4-hydroxy-4-(3-pyridyl)-butanoic acid	Alkaloids	down
1-(8-hydroxyoleoyl)-sn-glycero-3-phosphocholine	Lipids	up
3',4',5,5',6,7-Hexamethoxyflavone	Flavonoids	up
LysoPE 14:0	Lipids	down
α-Chaconine	Alkaloids	down
2,3-Dihydroxanthotoxin	Lignans and Coumarins	down
5-Hydroxytryptophol	Alkaloids	up
Leu-Glu-Val	Amino acids and derivatives	down
Gly-Tyr	Amino acids and derivatives	down
Tyr-Gly	Amino acids and derivatives	down
8-Methoxy-6,7-Methylenedioxycoumarin	Lignans and Coumarins	up
Rosmarinic acid methyl ester	Phenolic acids	down
tryptophy-lalanine	Amino acids and derivatives	down
Pipecolic acid	Alkaloids	up
3-Hydroxyurs-12-en-28-aldehyde (Ursolaldehyde)	Terpenoids	down
L-Tyrosine methyl ester	Amino acids and derivatives	down
N-Cinnamoylagmatine	Alkaloids	down
Tectoridin	Flavonoids	down
Sphaelactone A	Terpenoids	down
Ser-Trp	Amino acids and derivatives	down
N-p-Coumaroyllysine	Alkaloids	up
20,21-Dihydroxypregna-4,6-dien-3-one	Steroids	up
alismol	Terpenoids	up
4'-Methoxyacetophenone	Others	up
N-benzoyl-2-aminoethyl-β-D-glucopyranoside	Alkaloids	up
Lanosta-7,9(11),24-trien-3,26-diol (Ganoderol B)	Terpenoids	down
Lys-Ala	Amino acids and derivatives	down
Ser-Ile-Asp	Amino acids and derivatives	up
Betulin	Terpenoids	up
β-Chaconine	Alkaloids	up
Dimethoxysamin	Lignans and Coumarins	down
Monocrotaline N-oxide	Alkaloids	up

Thr-Trp	Amino acids and derivatives	down
Erigeside I	Others	down
γ-Glu-Tyr	Amino acids and derivatives	up
3-Ethynylbenzaldehyde	Others	down
4-[2-(1-methylethyl)aminoethyl]phenol	Alkaloids	up
Costunolide	Terpenoids	down
Ilicic acid	Terpenoids	up
1,3-Dihydroxyurs-12-en-28-oic acid (Kaneric acid)	Terpenoids	down
1-α-Linolenoyl-glycerol	Lipids	up
Virgatic acid	Terpenoids	up
Ipratropium	Alkaloids	up
L-Glutamyl-L-Tyrosine	Amino acids and derivatives	up
γ-Solanine	Alkaloids	up
PS(18:1/0:0)	Lipids	up
Pro-Leu-Asp	Amino acids and derivatives	up
5-Demethylnobiletin; 5-Hydroxy-6,7,8,3',4'-Pentamethoxyflavone	Flavonoids	up
4',6,7-Trihydroxyisoflavone	Flavonoids	up
3α,20β,21β-trihydroxy-16-oxo-serrat-14-en-24-oic acid	Terpenoids	up
methyl-4,5-diacetyloxy-3-hydroxy-6-[4-(hydroxymethyl)phenoxy]oxane-2-carboxylate	Phenolic acids	down
Val-Ile-Val	Amino acids and derivatives	down
Ile-Val-Trp	Amino acids and derivatives	down
Phe-Ala	Amino acids and derivatives	up
15(R)-Hydroxylinoleic Acid	Lipids	up
1,6-Dihydroxy-4(14)-eudesmene	Terpenoids	up
Ethylbutylacetylaminopropionate	Alkaloids	down
1-(sn-Glycero-3-phospho)-1D-myo-inositol	Others	up
Acetosyringone	Others	down
Tryptophan	Amino acids and derivatives	down
β-Hydroxyisovaleric acid	Organic acids	up
N-Acetyl-L-glycine	Amino acids and derivatives	down
3,4-Dihydroxyphenylacetate	Phenolic acids	down
Homogentisate	Phenolic acids	down
Dihydroferulic Acid	Phenolic acids	down
19,20-DiHDPA	Lipids	up
Linolelaidic acid ethyl ester	Lipids	up
Neochlorogenic acid	Phenolic acids	down
Eicosadienoic acid	Lipids	up
(R)-2-Hydroxyisocaproate	Organic acids	down
Chlorogenate	Phenolic acids	down
Protocatechuic Acid Methyl Ester	Phenolic acids	down
Cryptochlorogenic acid (4-O-Caffeoylquinic acid)	Phenolic acids	down
Atractylenolide III	Terpenoids	down

Sphinganine 1-phosphate	Lipids	down
Solatriose	Others	up
Hexylitaconic acid	Organic acids	down
trans-Cinnamate	Phenolic acids	down
D-Erythrose 4-phosphate	Others	up
Thiamin diphosphate	Others	down
3-Hydroxy-24-methylene-9,19-cyclolanostan-26-oic acid (Ambolic acid)	Terpenoids	down
alpha-D-Glucosamine 1-phosphate	Others	up
4-Methylhippuric Acid	Amino acids and derivatives	up
L-Fucitol	Others	down
2-Decenedioic acid	Lipids	up
HexosylLPE18:2	Lipids	up
9-Hydroperoxy-9Z,11E-Octadecadienoic Acid	Lipids	up
14,15-Epoxyeicosa-5z-enoic acid	Lipids	down
6-Geranylnaringenin; Mimulone; Bonannione A	Flavonoids	up
N-(1-Deoxy-1-fructosyl)Asparagine	Amino acids and derivatives	down
Taurine	Organic acids	down
2,3-dinor-9S,11R,15S-trihydroxy-5Z,13E-prostadienoic acid	Lipids	up
3-Hydroxyolean-12-en-28-oic acid (Oleanolic acid)	Terpenoids	up
N-Carbomethoxy-L-phenylalanine	Amino acids and derivatives	down
Hydroxy ricinoleic acid	Lipids	up
(R)-3-Hydroxybutanoate	Organic acids	down
Sucrose-6-phosphate	Others	up
alpha,alpha'-Trehalose 6-phosphate	Others	up
N-Acetyl-L-aspartate	Amino acids and derivatives	down
1,6-anhydro-β-D-glucose	Others	down
Ferulate	Phenolic acids	down
Isoferulic Acid	Phenolic acids	down
Phelligridin J	Phenolic acids	up
9,10,11-Trihydroxy-12-octadecenoic acid	Lipids	up
3-O-Methylgallate	Phenolic acids	down
10-Hydroxydecanoic acid	Lipids	up
N-(1-Deoxy-1-fructosyl)Tryptophan	Amino acids and derivatives	down
tryptophan N-glucoside	Amino acids and derivatives	down
Glucosyl 7-methyl-3-methyleneoctane-1,2,6,7-tetraol	Terpenoids	down
3,4-Open loop-lupine-4(23),20(29)-diene-24-hydroxy-3-carboxylic acid	Terpenoids	down
Phenylpropanoate	Phenolic acids	down
8-(Beta-D-Glucopyranosyloxy)-2,7-Dimethyl-2,4-Decadienedioic Acid	Terpenoids	up
1'-Galactoyl Ferulic acid	Phenolic acids	up
Phelligridin A	Phenolic acids	up

4-O-galactopyranosylxylose	Others	down
Genistin	Flavonoids	down
DGMG 18:3(2n isomer)	Lipids	up
Eucalyptin (5-Hydroxy-7,4'-dimethoxy-6,8-dimethylflavone)	Flavonoids	up
13-KODE; (9Z,11E)-13-Oxooctadeca-9,11-dienoic acid	Lipids	up
6-Galloyl Isoascorbic acid	Others	down
Ricinoleic acid methyl ester	Lipids	up
Aloe emodin-1-O-glucoside	Quinones	down
2,19-Dihydroxy-3-oxoursa-1,12-dien-28-oic acid (Fupenzic acid)	Terpenoids	up
Salidroside	Phenolic acids	up
6-Galloyl Ascorbic acid	Others	down
Keto-Deoxy-Nonulonic acid	Lipids	down
Terephthalate	Phenolic acids	down
5,7-Dihydroxy-1(3H)-isobenzofuranone	Others	down
2-Hydroxy-4-methyl-3-tridecanoyloxypentanoic acid methyl ester	Lipids	up
1-O-Vanilloyl-D-Glucose	Phenolic acids	up
9-Hydroxy-10,12,15-octadecatrienoic acid	Lipids	up
Cyrtominetin	Flavonoids	down
Glyceraldehyde	Others	up
Glucosyl 5,8-dihydroxy-2,6-dimethylocta-2,6-dienoic acid	Terpenoids	down
3-Hydroxydecanedioic acid	Lipids	up
Olivil-4'-O-glucoside	Lignans and Coumarins	up
10-Hydroxy-2-decenoic acid	Lipids	up
alpha-Oxo-benzeneacetic acid	Phenolic acids	down
Syringic acid 4-O-rhamnoside	Phenolic acids	up
Orcinol glucoside	Phenolic acids	down
3-Hydroxy-5-Methylphenol-1-O-Glucoside	Others	down
Reynosin	Terpenoids	down
Asp-Glu-Tyr	Amino acids and derivatives	down
orthosphenic acid	Terpenoids	down
2,4,6,4'-Tetrahydroxy-stilbene-2-O-glucoside	Others	down
3-Carboxy-4-Methyl-5-Propyl-2-Furanpropionic Acid	Organic acids	down
8-Hydroxyguanosine	Nucleotides and derivatives	up
trans-2-Hydroxycinnamate	Phenolic acids	down
3-Hydroxycinnamic Acid	Phenolic acids	down
3-Hydroxyurs-12-en-28-oic acid (3-Epiursolic acid)	Terpenoids	down
Urolithin D	Flavonoids	down
Glucosyl 2-Hydroxy-4-Methylpentanoic Acid	Organic acids	down
Danshensu; Salvianic Acid A	Phenolic acids	up
6-O-Feruloyl-glucose	Phenolic acids	down

Lucyoside F	Terpenoids	down
Malic acid-1-O-glucopyranoside	Organic acids	up
5-[(1S,2R,3R,5S)-3,5-dihydroxy-2-[(E,3S)-3-hydroxyoct-1-enyl]cyclopentyl]pentanoic acid	Lipids	up
p-Hydroxyphenyl 6-O-(E)-caffeoyl-β-D-allopyranoside	Phenolic acids	up
4-Quinolinecarboxylic acid	Alkaloids	up
Lactate	Organic acids	down
Oxalate	Organic acids	down
Methylprotocatechuate 4-(6''-acetylglucoside)	Phenolic acids	up
2-Caffeoyl-L-tartaric acid (Caftaric acid)	Phenolic acids	up
16,23:16,30-Diepoxydammar-24-ene-3,20-diol (Jujubogenin)	Terpenoids	down
3,19-Dihydroxyurs-12-en-28-oic acid (Pomolic acid)	Terpenoids	down
(-)-5'-Methoxyisolariciresinol 3alpha-O-beta-glucopyranoside	Lignans and Coumarins	down
Resveratrol-3,5-di-O-glucoside	Others	down

Table S5 IH-M VS IH-G analysis of differential metabolites results

Compounds	Class I	Type
inonophenol C	Phenolic acids	down
Deoxycytidine	Nucleotides and derivatives	down
Tryptamine	Alkaloids	down
LysoPE 16:1(2n isomer)	Lipids	up
Jasmone	Others	up
Dibutyl sebacate	Lipids	down
LysoPC 18:0	Lipids	down
LysoPC 17:1	Lipids	up
2-Acetyl-3-ethylpyrazine	Alkaloids	up
lancilactone A	Terpenoids	down
(2S,4R)-4-(Beta-D-Glucopyranosyloxy)-2-Pentanol	Others	down
5'-Deoxy-5'-fluoroadenosine	Others	down
Benzamide	Alkaloids	down
Oxypurinol	Nucleotides and derivatives	down
Cytidine	Nucleotides and derivatives	down
2-Ethynylbenzaldehyde	Others	down
Estrane-3,17-diol	Steroids	up
LysoPE 17:1(2n isomer)	Lipids	up
Sesamin	Lignans and Coumarins	down
cis-Zeatin riboside	Alkaloids	down
8-Azaguanine	Nucleotides and derivatives	down
Isoguanosine	Nucleotides and derivatives	down
Deoxyadenosine	Nucleotides and derivatives	down

L-Homocystine	Amino acids and derivatives	down
4-Aminobutanoate	Organic acids	down
5-Ribofuranosylnicotinamide	Nucleotides and derivatives	down
Thymine	Nucleotides and derivatives	down
Cordycepin	Nucleotides and derivatives	down
Cadaverine	Alkaloids	down
Deoxyguanosine	Nucleotides and derivatives	down
Allopurinol	Nucleotides and derivatives	down
5'-Deoxyadenosine	Nucleotides and derivatives	down
S-Adenosyl-L-methionine	Amino acids and derivatives	down
Phenylethyl alcohol	Others	down
Inosine	Nucleotides and derivatives	down
LysoPE 18:0	Lipids	down
Phenethylamine	Alkaloids	down
1-O-Hexadecyl-sn-glycero-3-phosphocholine	Lipids	down
Linolenyl Alcohol	Others	up
2-Aminopurine	Nucleotides and derivatives	down
Metformin	Alkaloids	up
Guanosine	Nucleotides and derivatives	down
Deoxyinosine	Nucleotides and derivatives	down
Isocytosine	Nucleotides and derivatives	up
Epigoitrin	Others	up
Aerugidiol	Terpenoids	up
LysoPE 17:1	Lipids	up
Securinine	Alkaloids	up
Allosecurinine	Alkaloids	up
1-Arabinosyluracil	Nucleotides and derivatives	down
LysoPE 15:0	Lipids	down
4-carbamoyl-2-(octadeca-9,12-dienamido)butanoic acid	Lipids	up
3,9,25-Trihydroxy-24-hydroxymethylergosta-7,22-dien-6-one	Steroids	down
nardostachone	Terpenoids	down
5-methylfurfural	Others	down
Sarmentosin	Alkaloids	up
Skimmin (7-Hydroxycoumarin-7-O-glucoside)	Lignans and Coumarins	down
7-Hydroxy-4-chromone	Others	up
9-Oxooctadeca-10,12-Dienoic Acid	Lipids	down
N-Formyl nornicotine	Alkaloids	down
Cyclo(Pro-Leu)	Amino acids and derivatives	up
Cyclo(D-Leu-L-Pro)	Amino acids and derivatives	up
N6-methyladenosine	Nucleotides and derivatives	down
3-Hydroxyanthranilate	Alkaloids	down
7-phenylhept-2-en-1-ol	Others	up
Hypoxanthine	Nucleotides and derivatives	down
Davallialactone	Others	up

2,3,4-Trihydroxybutyl 6-O-(E)-caffeoyl-β-D-glucopyranoside	Phenolic acids	down
Guaianediol	Terpenoids	down
Aristolone	Terpenoids	down
(2E)-3-(1-Hydroxy-2,6,6-trimethyl-4-oxo-2-cyclohexen-1-yl)-2-propenoic acid	Terpenoids	up
24-Hydroxy-3-oxooleana-11,13(18)-dien-28-oic acid	Terpenoids	up
Gly-Val-Ala	Amino acids and derivatives	up
Carnosine	Amino acids and derivatives	down
L-Kynurenine	Alkaloids	down
Cyclo(Pro-Val)	Amino acids and derivatives	down
9-[(2S,3S,4S,5R)-3,4-dihydroxy-5-(hydroxymethyl)oxolan-2-yl]-1H-purin-6-one	Alkaloids	down
L-Homocysteinesulfinic acid	Amino acids and derivatives	down
Dihydroisopelletierine	Alkaloids	up
6-Hydroxy-2-[2-(3'-methoxy-4'-hydroxyphenylethyl)]chromone	Others	down
Centaureidin (5,7,3'-Trihydroxy-3,6,4'-Trimethoxyflavone)	Flavonoids	up
Ganoderenic Acid A	Terpenoids	up
Tangeretin	Flavonoids	down
3',4',5',5,7-Pentamethoxyflavone	Flavonoids	down
3-(1-Phenyl-2-methylprop-1-yl)-4-hydroxycoumarin	Lignans and Coumarins	down
Glutinosone	Terpenoids	down
Ganoderic Acid Sigma	Terpenoids	up
2-oxononadecanoic acid	Lipids	down
Septemlobin E	Terpenoids	down
5-Hydroxyquinoline	Alkaloids	up
Neocnidilide	Others	up
LysoPC 19:0	Lipids	down
1-(4-Hydroxy-3-methoxyphenyl)-7-phenylhept-4-en-3-one	Others	up
Magnograndiolide	Terpenoids	down
N-(1-Deoxy-1-fructosyl)isoleucine	Amino acids and derivatives	down
11,12-O-Isopropyfidenesolajiangxin F	Terpenoids	down
1,7-Bis-(4-hydroxyphenyl)-2,4,6-heptatrienone	Others	down
Leu-Glu-Ile	Amino acids and derivatives	down
L-Cystine	Amino acids and derivatives	up
N-(1-Deoxy-1-fructosyl)Valine	Amino acids and derivatives	down
(1,3)-1-Methyl-1,2,3,4-tetrahydro-β-carboline-3-carboxylic acid	Alkaloids	down
Decitabine; 5-Aza-2'-Deoxycytidine	Nucleotides and derivatives	down
Inoscavin A	Others	up
Ladanetin-6-O-β-D-glucoside	Flavonoids	up
Cyclo(D-Val-L-Pro)	Amino acids and derivatives	up
2-Hydroxy-2,3-dihydronaphthalene-1,4-dione	Quinones	up
Cyclo(Val-Ala)	Amino acids and derivatives	up
Eremofukinone	Terpenoids	down
6,7-Dimethoxy-2-(2-phenylethyl)chromone	Others	down
N-Hydroxyhexanoyl nornicotine glucoside	Alkaloids	up

His-Asp	Amino acids and derivatives	down
4-(2-aminoethenyl)phenol	Alkaloids	up
Ser-Lys	Amino acids and derivatives	up
cycloalliin	Amino acids and derivatives	up
2-(Acetylamino)-3-phenyl-2-propenoic acid	Alkaloids	up
Tyr-Leu	Amino acids and derivatives	up
Lys-Asn	Amino acids and derivatives	up
3',4',5,5',6,7-Hexamethoxyflavone	Flavonoids	down
Anhydro-β-rotunol	Terpenoids	down
LysoPE 14:0	Lipids	down
2,3-Dihydroxanthotoxin	Lignans and Coumarins	up
5-Hydroxytryptophol	Alkaloids	down
4,5-Dihydrovmifoliol	Terpenoids	up
8-Methoxy-6,7-Methylenedioxycoumarin	Lignans and Coumarins	down
Ergothioneine	Others	up
4-((6S)-6-(furan-3-yl)-3-(hydroxymethyl)piperidin-2-yl)-2-methylbutan-2-ol	Alkaloids	up
Indolin-2-one	Alkaloids	up
Dihydro-N-methylisopelletierine	Alkaloids	down
3-Hydroxylanosta-7,9(11),24-trien-26-oic acid (Ganoderic Acid Y)	Terpenoids	up
Sphaelactone A	Terpenoids	up
N-p-Coumaroyllysine	Alkaloids	down
Phelligridin F	Others	down
Fraxinellone	Others	up
alismol	Terpenoids	down
phomophyllin F	Terpenoids	up
4'-Methoxyacetophenone	Others	up
N-benzoyl-2-aminoethyl-β-D-glucopyranoside	Alkaloids	down
Lys-Ala	Amino acids and derivatives	up
beta-Rotunol	Terpenoids	down
4-Phenylbutyric acid	Organic acids	down
3-O-Methylellagic acid-4'-O-(5''-O-acetyl)arabinoside	Tannins	up
Betulin	Terpenoids	down
3-Hydroxyurs-5(6),12,18(19)-trien-28-oic acid (Uncargenin B)	Terpenoids	up
β-Chaconine	Alkaloids	down
Fukinolic acid	Tannins	down
4-Hydroxyaniline	Alkaloids	up
2-Aminophenol	Alkaloids	up
3-hydroxybenzaldehyde	Others	up
Thr-Trp	Amino acids and derivatives	up
4-[2-(1-methylethyl)aminoethyl]phenol	Alkaloids	down
N-Hydroxytryptamine	Alkaloids	down
Serotonin; 5-Hydroxytryptamine	Alkaloids	down
Capsidiol	Terpenoids	down
Ilicic acid	Terpenoids	down

p-Anisaldehyde	Others	up
Tamarixetin	Flavonoids	down
Ipratropium	Alkaloids	down
L-Glutamyl-L-Tyrosine	Amino acids and derivatives	down
γ-Solanine	Alkaloids	down
PS(18:1/0:0)	Lipids	down
Pro-Leu-Asp	Amino acids and derivatives	down
5-Demethylnobiletin; 5-Hydroxy-6,7,8,3',4'-Pentamethoxyflavone	Flavonoids	down
3α,20β,21β-trihydroxy-16-oxo-serrat-14-en-24-oic acid	Terpenoids	up
Ligularone	Terpenoids	up
Daminozide	Alkaloids	down
Phe-Ala	Amino acids and derivatives	down
15(R)-Hydroxylinoleic Acid	Lipids	up
1,6-Dihydroxy-4(14)-eudesmene	Terpenoids	down
Ethylbutylacetylaminopropionate	Alkaloids	up
dGMP	Nucleotides and derivatives	down
Guanosine 5'-monophosphate	Nucleotides and derivatives	down
3',5'-Cyclic AMP	Nucleotides and derivatives	down
Vestitol	Flavonoids	down
17-Hydroxylinolenic acid	Lipids	up
6-Hydroxyhexanoic acid	Organic acids	down
3-Phospho-D-glycerate	Organic acids	up
alpha-Hydroxylinoleic acid	Lipids	up
Xanthine	Nucleotides and derivatives	down
N6-(1,2-Dicarboxyethyl)-AMP	Nucleotides and derivatives	down
13(S)-HODE;13(S)-Hydroxyoctadeca-9Z,11E-dienoic acid	Lipids	up
9S-Hydroxy-10E,12Z-octadecadienoic acid	Lipids	up
Undecanedioic acid	Lipids	down
(7Z)-Hexadecenoic acid	Lipids	up
N-Fructosyl Pyroglutamate	Amino acids and derivatives	up
N-Acetylornithine	Amino acids and derivatives	up
9-(Arabinosyl)hypoxanthine	Nucleotides and derivatives	down
trans-Cinnamate	Phenolic acids	down
N-Acetyl-D-glucosamine 1-phosphate	Others	down
2(3H)-Benzothiazolone	Alkaloids	up
Guanosine 3'-phosphate	Alkaloids	down
1-(2,3-dihydroxypropoxy)-3-(((2-(dimethylamino)ethoxy)(hydroxy)phosphoryl)oxy)propan-2-yl palmitate	Lipids	down
Panose	Others	down
Raffinose	Others	down
Kaurenoic Acid	Terpenoids	down
Uridine	Nucleotides and derivatives	down
4-Hydroxyphthalic acid	Phenolic acids	up
D-Fructose 1,6-bisphosphate	Others	down

Pseudouridine	Nucleotides and derivatives	down
9-Hydroxy-13-oxo-10-octadecenoic Acid	Lipids	up
AICAR phosphate (Acadesine phosphate)	Nucleotides and derivatives	down
Sarcosine	Amino acids and derivatives	down
13S-Hydroxy-9Z,11E,15Z-octadecatrienoic acid	Lipids	up
HexosylLPE18:2	Lipids	up
3',5'-Cyclic GMP	Nucleotides and derivatives	down
glycoric acid	Others	down
9-Hydroperoxy-9Z,11E-Octadecadienoic Acid	Lipids	up
Eriodictyol chalcone	Flavonoids	down
2-Carboxy-3-hydroxy-A(1)-norlupan-20(29)-en-28-oic acid (Ceanothic acid)	Terpenoids	up
Phosphoenolpyruvate	Organic acids	up
Uridine 5'-monophosphate	Nucleotides and derivatives	down
D-Gal alpha 1→6D-Gal alpha 1→6D-Glucose	Others	down
Glyceraldehyde 3-phosphate	Organic acids	down
16-Hydroxy-24-methylene-3-oxolanosta-7,9(11)-diene-21-oic acid (Polyporenic acid C)	Terpenoids	down
(R)-Mandelate	Organic acids	down
6-Geranylnaringenin; Mimulone; Bonannione A	Flavonoids	up
L-Alanine	Amino acids and derivatives	down
2-Dodecenedioic acid	Lipids	down
alpha-D-Glucose 1,6-bisphosphate	Others	down
Maltotetraose	Others	down
9-Oxo-10E,12Z-octadecadienoic acid	Lipids	up
Rabdosia acid A	Lipids	up
N-Carbomethoxy-L-phenylalanine	Amino acids and derivatives	up
Hydroxy ricinoleic acid	Lipids	down
Xanthosine	Nucleotides and derivatives	down
O-Phospho-L-serine	Amino acids and derivatives	down
Laricitrin-3-O-(6''-acetyl)glucoside	Flavonoids	down
N-Acetyl-L-aspartate	Amino acids and derivatives	up
1,6-anhydro-β-D-glucose	Others	up
N6-(L-1,3-Dicarboxypropyl)-L-lysine	Amino acids and derivatives	down
Ferulate	Phenolic acids	up
Isoferulic Acid	Phenolic acids	up
3-Hydroxy-3-methylglutarate	Organic acids	down
Norartocarpetin	Flavonoids	down
Ethyl caffeate	Phenolic acids	down
9S-Hydroperoxy-10E,12Z-octadecadienoic acid	Lipids	up
Deoxyuridine	Nucleotides and derivatives	down
Prasterone	Steroids	down
9,10,11-Trihydroxy-12-octadecenoic acid	Lipids	down
3-O-Methylgallate	Phenolic acids	up
9,16-Dihydroxypalmitic acid	Lipids	down

N-(1-Deoxy-1-fructosyl)Tryptophan	Amino acids and derivatives	up
tryptophan N-glucoside	Amino acids and derivatives	up
Glucosyl 7-methyl-3-methyleneoctane-1,2,6,7-tetraol	Terpenoids	up
3-O-Acetylpinobanksin	Flavonoids	down
1-(2,3-dihydroxypropoxy)-3-(((2-(dimethylamino)ethoxy)(hydroxy)phosphoryl)oxy)propan-2-yl heptadecanoate	Lipids	down
(3-hydroxypropanoyl)-L-leucine	Amino acids and derivatives	down
Pyruvate	Organic acids	down
Feruloyltartaric acid (Fertaric acid)	Phenolic acids	down
dendrodolideK	Others	down
9,12,13-Trihydroxy-10,15-octadecadienoic acid	Lipids	down
2,3-Dihydroxyolean-12-en-28-oic acid (2-Hydroxyoleanolic acid)	Terpenoids	up
Phenylpropanoate	Phenolic acids	down
3-hydroxy-5-methoxybenzaldehyde	Phenolic acids	down
Phelligridin A	Phenolic acids	up
12(13)Ep-9-KODE	Lipids	down
N-Acetylneuraminate	Amino acids and derivatives	down
N-(1-Deoxy-1-fructosyl)Piperidic acid	Alkaloids	up
9-Hydroxy-12-oxo-15(Z)-octadecenoic acid	Lipids	down
Citrinin	Others	down
DGMG 18:3(2n isomer)	Lipids	up
4-Aminosalicylate	Phenolic acids	up
Eucalyptin (5-Hydroxy-7,4'-dimethoxy-6,8-dimethylflavone)	Flavonoids	down
Eriodictyol	Flavonoids	down
2,2'-(3-methylcyclohexane-1,1-diyl)diacetic acid	Organic acids	down
3-Guanidinopropanoate	Organic acids	down
Glucosyl 6,9-dihydroxydec-4-enoic acid	Terpenoids	down
13-KODE; (9Z,11E)-13-Oxooctadeca-9,11-dienoic acid	Lipids	down
Hydroperoxylinoleic acid	Lipids	down
Ricinoleic acid methyl ester	Lipids	up
Aflatoxin G1 9,10-epoxide	Others	down
3-Aminosalicylic acid	Phenolic acids	down
2,19-Dihydroxy-3-oxoursa-1,12-dien-28-oic acid (Fupenzic acid)	Terpenoids	down
Phomolide G	Others	down
Terephthalate	Phenolic acids	down
5,7-Dihydroxy-1(3H)-isobenzofuranone	Others	down
2-Hydroxy-4-methyl-3-tridecanoyloxypentanoic acid methyl ester	Lipids	up
N-acetyl-beta-alanine	Amino acids and derivatives	down
2-Oxoglutarate	Organic acids	down
5-hydroxy-anthraquinone-2-carboxylic acid	Quinones	up
9-Hydroxy-10,12,15-octadecatrienoic acid	Lipids	down
Glyceraldehyde	Others	down
Olivil-4'-O-glucoside	Lignans and Coumarins	down
10-Hydroxy-2-decenoic acid	Lipids	up

alpha-Oxo-benzeneacetic acid	Phenolic acids	up
Syringic acid 4-O-rhamnoside	Phenolic acids	down
Prolylhydroxyproline	Amino acids and derivatives	down
4-hydroxy-4-(2-hydroxyphenyl)butanoic acid	Others	up
Dihydroartemisinin	Terpenoids	down
10-hydroxymajoroside	Terpenoids	up
orthosphenic acid	Terpenoids	down
N-Acetyl-6-hydroxy-L-tryptophan	Amino acids and derivatives	down
Porrigenic acid	Lipids	up
8-Hydroxyguanosine	Nucleotides and derivatives	down
trans-2-Hydroxycinnamate	Phenolic acids	up
3-Hydroxycinnamic Acid	Phenolic acids	up
Danshensu; Salvianic Acid A	Phenolic acids	down
Malic acid-1-O-glucopyranoside	Organic acids	down
5-[(1S,2R,3R,5S)-3,5-dihydroxy-2-[(E,3S)-3-hydroxyoct-1-enyl]cyclopentyl]pentanoic acid	Lipids	down
Petasiphenone	Phenolic acids	down
(4-(2-oxopropoxy)phenyl)-L-alanine	Amino acids and derivatives	up
p-Hydroxyphenyl 6-O-(E)-caffeoyl-β-D-allopyranoside	Phenolic acids	down
3,23-Dihydroxy-30-noroleana-12,20(29)-dien-28-oic acid (30-Norhederagenin)	Terpenoids	up
4-Quinolinecarboxylic acid	Alkaloids	down
3-O-(3-Methoxy-4-hydroxybenzoyl)-4-O-caffeoylquinic acid	Phenolic acids	down
2-Aldehydo-A(1)-norlup-20(29)-en-27,28-dioic acid (Zizyberanal acid)	Terpenoids	up
3-Hydroxyurs-12-en-28-oic acid (Ursolic acid)	Terpenoids	up
3-Hydroxy-9,19-cyclolanost-24-en-26-oic acid (Mangiferolic acid)	Terpenoids	up
Methylprotocatechuate 4-(6''-acetylglucoside)	Phenolic acids	down
Aminomalonate	Amino acids and derivatives	down
2-Caffeoyl-L-tartaric acid (Caftaric acid)	Phenolic acids	down
3-(3,4-dihydroxyphenyl)propanoic acid glucoside	Phenolic acids	up

Table S6 IH-M VS IH-J analysis of differential metabolites results

**Compounds**	**Class I**	**Type**
3,3'-methylene-bis [6-(3,4-dihydroxystyryl)-4-hydroxy-2H-pyran-2-one	Others	down
inonophenol C	Phenolic acids	down
hispindic acid B	Terpenoids	down
6,7-dimethoxy-1-methylisoquinoline	Alkaloids	down
Vasicine	Alkaloids	down
5-Methoxyindoleacetate	Alkaloids	down
Deoxycytidine	Nucleotides and derivatives	down
Tuliposide D	Others	down
3-(1H-imidazol-5-yl)-2-(trimethylammonio)propanoate	Alkaloids	down
O-Acetyl-L-carnitine	Alkaloids	down

Tryptamine	Alkaloids	down
LysoPC 18:1	Lipids	up
LysoPE 16:1(2n isomer)	Lipids	up
Glycidyl Linoleate	Lipids	up
LysoPC 17:2(2n isomer)	Lipids	up
Sessilifol O	Terpenoids	down
LysoPC 18:0	Lipids	down
Harman-3-carboxylic acid	Alkaloids	down
LysoPC 17:1	Lipids	up
(S)-14-Methylhexadecanoic acid	Lipids	down
Vitetrifolin H	Terpenoids	up
LysoPE 18:2	Lipids	up
LysoPC 17:2	Lipids	up
2-Linoleoylglycerol	Lipids	up
2-Acetyl-3-ethylpyrazine	Alkaloids	up
Methyl linolenate	Lipids	down
(2S,4R)-4-(Beta-D-Glucopyranosyloxy)-2-Pentanol	Others	down
5'-Deoxy-5'-fluoroadenosine	Others	down
L-Tryptophanamide	Amino acids and derivatives	down
3-amino-2-naphthoic acid	Alkaloids	down
naphthisoxazol A	Alkaloids	down
3-Indoleacrylic acid	Alkaloids	down
2-methyl-1-nitronaphthalene	Others	down
Pyran[3,4-b]indole-2-ketone	Alkaloids	down
(Oxiran-2-yl)methyl octadeca-9,12-dienoate	Lipids	up
His-Asp-Ser	Amino acids and derivatives	up
2-(Dimethylamino)guanosine	Nucleotides and derivatives	down
LysoPC 18:1(2n isomer)	Lipids	up
Benzamide	Alkaloids	down
Oxypurinol	Nucleotides and derivatives	down
Octadeca-9,12,15-trienoic acid	Lipids	up
Punicic acid	Lipids	up
LysoPE 18:4	Lipids	up
2-Ethynylbenzaldehyde	Others	down
LysoPC 16:1(2n isomer)	Lipids	up
Oxyphyllanene D	Terpenoids	up
3H-Naphth[1,8ab]oxirene-8-acetic acid	Terpenoids	up
3-Hydroxyhexadecanoic acid	Lipids	up
Dendronobilin I	Terpenoids	up
LysoPE 18:0(2n isomer)	Lipids	up
Cyclo(Pro-Pro)	Amino acids and derivatives	up
Gorlic acid	Lipids	up
Estrane-3,17-diol	Steroids	up
Riboflavin	Others	down

LysoPE 17:1(2n isomer)	Lipids	up
Glu-Ile-Val	Amino acids and derivatives	up
Sesamin	Lignans and Coumarins	down
cis-Zeatin riboside	Alkaloids	down
Octadeca-2,9,12,15-tetraen-1-ol	Others	up
9R-hydroxy-10E,12Z-octadecadienoic acid	Lipids	up
LysoPE 18:3(2n isomer)	Lipids	up
8-Azaguanine	Nucleotides and derivatives	down
LysoPE 18:1(2n isomer)	Lipids	up
2,3-Dihydroxypropyl 2-[(octadec-9-enoyl)amino]ethyl hydrogen phosphate	Lipids	up
Costunolide Derivative	Terpenoids	up
LysoPE 18:1	Lipids	up
Isoguanosine	Nucleotides and derivatives	down
Deoxyadenosine	Nucleotides and derivatives	down
Dibutylamine	Alkaloids	down
Acetoxy-[6]-gingerol	Phenolic acids	up
Methyl-[8]-gingerol	Phenolic acids	up
L-Homocystine	Amino acids and derivatives	down
Aflatrem	Alkaloids	up
L-isoleucyl-L-aspartyl-L-isoleucine	Amino acids and derivatives	up
19,20-Epoxycytochalasin D	Others	up
L-Glutamine	Amino acids and derivatives	up
L-Lysine	Amino acids and derivatives	up
3-(triazan-2-yl)propyl L-prolinate	Amino acids and derivatives	up
NG,NG-Dimethyl-L-arginine	Amino acids and derivatives	up
ethyl-L-arginine	Amino acids and derivatives	up
N,N'-Dimethylarginine;SDMA	Amino acids and derivatives	up
L-Ornithine	Amino acids and derivatives	up
N-(3-Indolylacetyl)-L-alanine	Amino acids and derivatives	down
2-Methylserine	Alkaloids	down
N',N'',N'''-p-Coumaroyl-cinnamoyl-caffeoyl spermidine	Alkaloids	up
4-hydroxy-2-oxo-1,2-dihydroquinoline-3-carboxylic acid	Alkaloids	down
4-Aminobutanoate	Organic acids	down
5-Ribofuranosylnicotinamide	Nucleotides and derivatives	down
His-Glu-Leu	Amino acids and derivatives	down
Glycyl-leucine	Amino acids and derivatives	down
Thymine	Nucleotides and derivatives	down
N(6)-OH-Me-Adenosine	Nucleotides and derivatives	down
Cordycepin	Nucleotides and derivatives	down
3-Indolepropionic acid	Alkaloids	down
4-amino-5-oxo-5-(pentylamino)pentanoic acid	Amino acids and derivatives	down
Deoxyguanosine	Nucleotides and derivatives	down
Pro-Ser-Glu	Amino acids and derivatives	up
S-Adenosyl-L-homocysteine	Amino acids and derivatives	down

A-hydroxyquinoline	Alkaloids	down
Allopurinol	Nucleotides and derivatives	down
N6-(2-Hydroxyethyl)adenosine	Nucleotides and derivatives	down
5'-Deoxyadenosine	Nucleotides and derivatives	down
3,5-Dihydro-2H-Furo[3,2-C]Quinolin-4-One	Alkaloids	down
Butyl beta-D-glucoside	Others	up
S-Adenosyl-L-methionine	Amino acids and derivatives	down
NAD+	Nucleotides and derivatives	down
Homoproline	Amino acids and derivatives	up
Phenylethyl alcohol	Others	down
L-Cystathionine	Amino acids and derivatives	up
Inosine	Nucleotides and derivatives	down
LysoPE 18:0	Lipids	down
Pro-Pro-Tyr	Amino acids and derivatives	down
Phenethylamine	Alkaloids	down
1-O-Hexadecyl-sn-glycero-3-phosphocholine	Lipids	down
Linolenyl Alcohol	Others	up
2-Aminopurine	Nucleotides and derivatives	down
L-Glycyl-L-isoleucine	Amino acids and derivatives	down
Metformin	Alkaloids	up
Guanosine	Nucleotides and derivatives	down
Deoxyinosine	Nucleotides and derivatives	down
2-Piperidone	Alkaloids	up
N6,N6-Dimethyladenosine	Amino acids and derivatives	up
4,5-Epoxy-14-hydroxy sinomenine N-oxide	Alkaloids	up
Isocytosine	Nucleotides and derivatives	up
Homoarginine	Amino acids and derivatives	up
Epigoitrin	Others	up
Aerugidiol	Terpenoids	up
1-Aminocyclopentanecarboxylate	Amino acids and derivatives	up
LysoPE 17:1	Lipids	up
CycloPro-Arg	Amino acids and derivatives	down
Asn-Gln-Cys	Amino acids and derivatives	up
Choline phosphate	Alkaloids	down
Phe-Thr	Amino acids and derivatives	down
N-Caffeoyltyramine	Alkaloids	down
N-Acetyl-DL-tryptophan	Amino acids and derivatives	up
LysoPE 18:3	Lipids	up
praeroside I	Lignans and Coumarins	up
Securinine	Alkaloids	up
Allosecurinine	Alkaloids	up
L-Alanyl-L-tryptophan	Amino acids and derivatives	down
N-Methyl-Trans-4-Hydroxy-L-Proline	Amino acids and derivatives	down
1-Arabinosyluracil	Nucleotides and derivatives	down

CDP-choline	Nucleotides and derivatives	down
3-Hydroxy-L-phenylalanine	Amino acids and derivatives	up
10-Formyltetrahydrofolate	Alkaloids	up
5,6-DiHETrE[(±)5,6-dihydroxy-8Z,11Z,14Z-eicosatrienoic acid]	Lipids	up
LysoPE 15:0	Lipids	down
4-carbamoyl-2-(octadeca-9,12-dienamido)butanoic acid	Lipids	up
3,9,25-Trihydroxy-24-hydroxymethylergosta-7,22-dien-6-one	Steroids	down
21β-Hydroxy-27-nor-C(14a)-homogammacera-14(15)-ene-3,16-dione	Terpenoids	up
Septemlobin D	Terpenoids	up
4-Hydroxypipecolic acid	Alkaloids	down
Nardosinone	Terpenoids	up
5-methylfurfural	Others	down
N-Methyl-α-aminoisobutyric acid	Amino acids and derivatives	up
Glu-Ser	Amino acids and derivatives	up
L-Lysine-Butanoic Acid	Amino acids and derivatives	up
γ-Glu-Trp	Amino acids and derivatives	down
Indole-3-carboxaldehyde	Alkaloids	down
Sarmentosin	Alkaloids	down
3-(Pyrazol-1-yl)-L-alanine	Amino acids and derivatives	down
Retronecine	Alkaloids	down
His-Tyr	Amino acids and derivatives	down
Skimmin (7-Hydroxycoumarin-7-O-glucoside)	Lignans and Coumarins	down
Isocurcumenol	Terpenoids	up
LysoPE 20:4	Lipids	up
LysoPC 19:1	Lipids	up
N-Formyl nornicotine	Alkaloids	down
N6-methyladenosine	Nucleotides and derivatives	down
3-Hydroxyanthranilate	Alkaloids	down
N7-Methylguanosine	Nucleotides and derivatives	down
N-Hexanoyl nornicotine	Alkaloids	up
7-phenylhept-2-en-1-ol	Others	up
9-hydroxy-4,8-dimethylnona-3,7-dienal	Terpenoids	up
Asp-Ile-Leu	Amino acids and derivatives	up
L-Histidine	Amino acids and derivatives	down
Hypoxanthine	Nucleotides and derivatives	down
Ala-Ile-Ala	Amino acids and derivatives	up
Davallialactone	Others	up
2,3,4-Trihydroxybutyl 6-O-(E)-caffeoyl-β-D-glucopyranoside	Phenolic acids	down
Guaianediol	Terpenoids	down
2-Oxo-3,4-dihydro-1H-quinoline-3-carboxylic acid	Alkaloids	down
5α,6α,7β,10β-11α,13-dihydro-4(15)-eudesmene-12,6-olide	Terpenoids	up
Tsoongiodendroonolide		
(2E)-3-(1-Hydroxy-2,6,6-trimethyl-4-oxo-2-cyclohexen-1-yl)-2-propenoic acid	Terpenoids	up
Brevianamide F	Alkaloids	up

24-Hydroxy-3-oxooleana-11,13(18)-dien-28-oic acid	Terpenoids	up
m-Aminophenylacetylene	Alkaloids	down
Carnosine	Amino acids and derivatives	down
L-Kynurenine	Alkaloids	down
Pro-Gly-Val	Amino acids and derivatives	up
Cyclo(Pro-Val)	Amino acids and derivatives	down
9-[(2S,3S,4S,5R)-3,4-dihydroxy-5-(hydroxymethyl)oxolan-2-yl]-1H-purin-6-one	Alkaloids	down
L-Homocysteinesulfinic acid	Amino acids and derivatives	down
Vanillylamine	Alkaloids	up
[4]-Shogaol	Phenolic acids	up
6-Hydroxy-2-[2-(3'-methoxy-4'-hydroxyphenylethyl)]chromone	Others	down
12,15-dioxo-selina-4,11-dine	Terpenoids	up
LysoPC 14:0	Lipids	down
LysoPE 15:0(2n isomer)	Lipids	down
Ganoderenic Acid A	Terpenoids	up
Tangeretin	Flavonoids	down
3',4',5',5,7-Pentamethoxyflavone	Flavonoids	down
Ganoderic Acid Sigma	Terpenoids	up
Asp-Ile-Gly	Amino acids and derivatives	up
2-oxononadecanoic acid	Lipids	down
5-Hydroxyquinoline	Alkaloids	up
Neocnidilide	Others	up
LysoPC 18:0(2n isomer)	Lipids	down
L-Cyclopentylglycine	Amino acids and derivatives	up
LysoPC 19:0	Lipids	down
1-(4-Hydroxy-3-methoxyphenyl)-7-phenylhept-4-en-3-one	Others	up
Magnograndiolide	Terpenoids	down
1,7-Bis-(4-hydroxyphenyl)-2,4,6-heptatrienone	Others	down
Met-Asn	Amino acids and derivatives	down
Leu-Glu-Ile	Amino acids and derivatives	down
N-(1-Deoxy-1-fructosyl)Valine	Amino acids and derivatives	down
Abscisic acid D-glucopyranosyl ester	Terpenoids	up
Pyrroline	Alkaloids	up
Val-His	Amino acids and derivatives	up
Nicotinic Acid Methyl Ester(Methyl Nicotinate)	Alkaloids	down
7-O-Methylaloesin	Flavonoids	down
Decitabine; 5-Aza-2'-Deoxycytidine	Nucleotides and derivatives	down
Inoscavin A	Others	up
Cyclo(D-Val-L-Pro)	Amino acids and derivatives	up
3S,4R,5S,8R,10R-tetrahydroperezinone	Terpenoids	up
4-Hydroxy-β-bulnesene	Others	up
Caffeoylcholine	Alkaloids	up
Cyclo(Val-Ala)	Amino acids and derivatives	up

5-methyl 1-propyl L-glutamate	Amino acids and derivatives	down
Isorhamnetin 3-glucuronide	Flavonoids	up
Ser-Gly-Leu	Amino acids and derivatives	down
N-Hydroxyhexanoyl nornicotine glucoside	Alkaloids	up
4-(2-aminoethenyl)phenol	Alkaloids	up
Ser-Lys	Amino acids and derivatives	up
2-(Acetylamino)-3-phenyl-2-propenoic acid	Alkaloids	up
5-Acetylsalicylic acid	Phenolic acids	down
Tyr-Leu	Amino acids and derivatives	up
4-hydroxy-4-(3-pyridyl)-butanoic acid	Alkaloids	down
Lys-Asn	Amino acids and derivatives	up
1-(8-hydroxyoleoyl)-sn-glycero-3-phosphocholine	Lipids	up
3',4',5,5',6,7-Hexamethoxyflavone	Flavonoids	down
Anhydro-β-rotunol	Terpenoids	down
LysoPE 14:0	Lipids	down
α-Chaconine	Alkaloids	down
Leu-Glu-Val	Amino acids and derivatives	down
Gly-Tyr	Amino acids and derivatives	down
Tyr-Gly	Amino acids and derivatives	down
4,5-Dihydrovmifoliol	Terpenoids	up
Rosmarinic acid methyl ester	Phenolic acids	down
tryptophy-lalanine	Amino acids and derivatives	down
Pipecolic acid	Alkaloids	up
Ergothioneine	Others	up
Ile-Glu-Val	Amino acids and derivatives	up
4-((6S)-6-(furan-3-yl)-3-(hydroxymethyl)piperidin-2-yl)-2-methylbutan-2-ol	Alkaloids	up
Indolin-2-one	Alkaloids	up
Dihydro-N-methylisopelletierine	Alkaloids	down
3-Hydroxyurs-12-en-28-aldehyde (Ursolaldehyde)	Terpenoids	down
L-Tyrosine methyl ester	Amino acids and derivatives	down
N-Cinnamoylagmatine	Alkaloids	down
Methyl 4-caffeoylquinate	Phenolic acids	down
3-Hydroxylanosta-7,9(11),24-trien-26-oic acid (Ganoderic Acid Y)	Terpenoids	up
Tectoridin	Flavonoids	down
Ser-Trp	Amino acids and derivatives	down
20,21-Dihydroxypregna-4,6-dien-3-one	Steroids	up
Phelligridin F	Others	down
Fraxinellone	Others	up
phomophyllin F	Terpenoids	up
4'-Methoxyacetophenone	Others	up
Lanosta-7,9(11),24-trien-3,26-diol (Ganoderol B)	Terpenoids	down
beta-Rotunol	Terpenoids	down
4-Phenylbutyric acid	Organic acids	down
Ser-Ile-Asp	Amino acids and derivatives	up

3-O-Methylellagic acid-4'-O-(5''-O-acetyl)arabinoside	Tannins	up
3-Hydroxyurs-5(6),12,18(19)-trien-28-oic acid (Uncargenin B)	Terpenoids	up
Fukinolic acid	Tannins	down
4-Hydroxyaniline	Alkaloids	up
2-Aminophenol	Alkaloids	up
3-hydroxybenzaldehyde	Others	up
Dimethoxysamin	Lignans and Coumarins	down
Monocrotaline N-oxide	Alkaloids	up
Erigeside I	Others	down
γ-Glu-Tyr	Amino acids and derivatives	up
3-Ethynylbenzaldehyde	Others	down
N-Hydroxytryptamine	Alkaloids	down
Serotonin; 5-Hydroxytryptamine	Alkaloids	down
Costunolide	Terpenoids	down
p-Anisaldehyde	Others	up
1,3-Dihydroxyurs-12-en-28-oic acid (Kaneric acid)	Terpenoids	down
1-α-Linolenoyl-glycerol	Lipids	up
Tamarixetin	Flavonoids	down
Virgatic acid	Terpenoids	up
PS(18:1/0:0)	Lipids	down
4',6,7-Trihydroxyisoflavone	Flavonoids	up
3α,20β,21β-trihydroxy-16-oxo-serrat-14-en-24-oic acid	Terpenoids	up
methyl-4,5-diacetyloxy-3-hydroxy-6-[4-(hydroxymethyl)phenoxy]oxane-2-carboxylate	Phenolic acids	down
Ligularone	Terpenoids	up
Val-Ile-Val	Amino acids and derivatives	down
Daminozide	Alkaloids	down
Ile-Val-Trp	Amino acids and derivatives	down
15(R)-Hydroxylinoleic Acid	Lipids	up
1,6-Dihydroxy-4(14)-eudesmene	Terpenoids	down
Glu-Asn	Amino acids and derivatives	down
Vestitol	Flavonoids	down
FAD	Nucleotides and derivatives	down
12,13-Epoxy-9-Octadecenoic Acid	Lipids	up
(9Z,11E)-(13S)-13-Hydroperoxyoctadeca-9,11-dienoic acid	Lipids	up
17-Hydroxylinolenic acid	Lipids	up
Acetosyringone	Others	down
Tryptophan	Amino acids and derivatives	down
Pimelate	Lipids	up
2-Propylsuccinic acid	Organic acids	up
(9S)-Hydroxyoctadecadienoic acid	Lipids	up
6-Hydroxyhexanoic acid	Organic acids	down
Phenylalanyltyrosine	Amino acids and derivatives	down
β-Hydroxyisovaleric acid	Organic acids	up

1,14-Tetradecanedioic Acid	Lipids	down
N-Acetyl-L-glycine	Amino acids and derivatives	down
3,4-Dihydroxyphenylacetate	Phenolic acids	down
Homogentisate	Phenolic acids	down
Dihydroferulic Acid	Phenolic acids	down
3-Phospho-D-glycerate	Organic acids	up
alpha-Hydroxylinoleic acid	Lipids	up
9(10)-EpOME;(9R,10S)-(12Z)-9,10-Epoxyoctadecenoic acid	Lipids	up
Xanthine	Nucleotides and derivatives	down
2,2-Dimethylsuccinic acid	Organic acids	up
19,20-DiHDPA	Lipids	up
N6-(1,2-Dicarboxyethyl)-AMP	Nucleotides and derivatives	down
(9E)-Octadecenoic acid	Lipids	up
13(S)-HODE;13(S)-Hydroxyoctadeca-9Z,11E-dienoic acid	Lipids	up
9S-Hydroxy-10E,12Z-octadecadienoic acid	Lipids	up
Neochlorogenic acid	Phenolic acids	down
Undecanedioic acid	Lipids	down
(R)-2-Hydroxyisocaproate	Organic acids	down
(7Z)-Hexadecenoic acid	Lipids	up
Chlorogenate	Phenolic acids	down
12-Hydroxyoctadecanoic acid	Lipids	down
Cryptochlorogenic acid (4-O-Caffeoylquinic acid)	Phenolic acids	down
Atractylenolide III	Terpenoids	down
1-(2,3-dihydroxypropoxy)-3-(((2-(dimethylamino)ethoxy)(hydroxy)phosphoryl)oxy)propan-2-yl (E)-hexadec-9-enoate	Lipids	up
Sphinganine 1-phosphate	Lipids	down
Guanidinoacetate	Alkaloids	down
Solatriose	Others	up
N-Acetylornithine	Amino acids and derivatives	up
Hexylitaconic acid	Organic acids	down
9-(Arabinosyl)hypoxanthine	Nucleotides and derivatives	down
trans-Cinnamate	Phenolic acids	down
D-Erythrose 4-phosphate	Others	up
Thiamin diphosphate	Others	down
N-Acetyl-D-glucosamine 1-phosphate	Others	down
1-(2,3-dihydroxypropoxy)-3-(((2-(dimethylamino)ethoxy)(hydroxy)phosphoryl)oxy)propan-2-yl palmitate	Lipids	down
Panose	Others	down
Raffinose	Others	down
Kaurenoic Acid	Terpenoids	down
Uridine	Nucleotides and derivatives	down
4-Hydroxyphthalic acid	Phenolic acids	up
D-Fructose 1,6-bisphosphate	Others	down

alpha-Isopropylmalate	Organic acids	down
3-Hydroxy-24-methylene-9,19-cyclolanostan-26-oic acid (Ambolic acid)	Terpenoids	down
UDP-glucuronate	Nucleotides and derivatives	down
Phloretate	Phenolic acids	down
9-Hydroxy-13-oxo-10-octadecenoic Acid	Lipids	up
AICAR phosphate (Acadesine phosphate)	Nucleotides and derivatives	down
Sarcosine	Amino acids and derivatives	down
13S-Hydroxy-9Z,11E,15Z-octadecatrienoic acid	Lipids	up
2-Decenedioic acid	Lipids	up
Aloesaponol III	Quinones	down
3-Hydroxymandelate	Phenolic acids	down
HexosylLPE18:2	Lipids	up
3',5'-Cyclic GMP	Nucleotides and derivatives	down
glycoric acid	Others	down
9-Hydroperoxy-9Z,11E-Octadecadienoic Acid	Lipids	up
Eriodictyol chalcone	Flavonoids	down
14,15-Epoxyeicosa-5z-enoic acid	Lipids	down
2-Carboxy-3-hydroxy-A(1)-norlupan-20(29)-en-28-oic acid (Ceanothic acid)	Terpenoids	up
Phosphoenolpyruvate	Organic acids	up
Uridine 5'-monophosphate	Nucleotides and derivatives	down
2-Hydroxy-3-oxopropanoate	Organic acids	down
Hydroxypyruvic acid	Organic acids	down
2,3-Dihydro-1,4-naphthoquinone	Quinones	up
(R)-Mandelate	Organic acids	down
6-Geranylnaringenin; Mimulone; Bonannione A	Flavonoids	up
5-HEPE	Lipids	up
L-Alanine	Amino acids and derivatives	down
2-Dodecenedioic acid	Lipids	down
2-Hydroxy-4-methylpentanoic acid	Organic acids	down
Shikimic Acid-3-Phosphate	Organic acids	down
Taurine	Organic acids	down
Maltotetraose	Others	down
9-Oxo-10E,12Z-octadecadienoic acid	Lipids	up
2,3-dinor-9S,11R,15S-trihydroxy-5Z,13E-prostadienoic acid	Lipids	up
3-Hydroxyolean-12-en-28-oic acid (Oleanolic acid)	Terpenoids	up
Rabdosia acid A	Lipids	up
Hydroxy ricinoleic acid	Lipids	down
(R)-3-Hydroxybutanoate	Organic acids	down
Xanthosine	Nucleotides and derivatives	down
O-Phospho-L-serine	Amino acids and derivatives	down
Laricitrin-3-O-(6''-acetyl)glucoside	Flavonoids	down
D-4'-Phosphopantothenate	Lipids	down
(S)-Mandelate	Phenolic acids	down
N6-(L-1,3-Dicarboxypropyl)-L-lysine	Amino acids and derivatives	down

3-Hydroxy-3-methylglutarate	Organic acids	down
Phelligridin J	Phenolic acids	up
Norartocarpetin	Flavonoids	down
Ethyl caffeate	Phenolic acids	down
9S-Hydroperoxy-10E,12Z-octadecadienoic acid	Lipids	up
Deoxyuridine	Nucleotides and derivatives	down
Prasterone	Steroids	down
9,10,11-Trihydroxy-12-octadecenoic acid	Lipids	up
10-Hydroxydecanoic acid	Lipids	up
9,16-Dihydroxypalmitic acid	Lipids	down
3-O-Acetylpinobanksin	Flavonoids	down
triptonoterpene	Terpenoids	up
(3-hydroxypropanoyl)-L-leucine	Amino acids and derivatives	down
(E)-12-hydroxydodec-2-enoic acid	Lipids	up
3,4-Open loop-lupine-4(23),20(29)-diene-24-hydroxy-3-carboxylic acid	Terpenoids	down
dendrodolideK	Others	down
2,3-Dihydroxyolean-12-en-28-oic acid (2-Hydroxyoleanolic acid)	Terpenoids	up
Phenylpropanoate	Phenolic acids	down
8-(Beta-D-Glucopyranosyloxy)-2,7-Dimethyl-2,4-Decadienedioic Acid	Terpenoids	up
1'-Galactoyl Ferulic acid	Phenolic acids	up
3-hydroxy-5-methoxybenzaldehyde	Phenolic acids	down
Phelligridin A	Phenolic acids	up
4-O-galactopyranosylxylose	Others	down
N-Acetylneuraminate	Amino acids and derivatives	down
Genistin	Flavonoids	down
2-(2,3-dihydroxypropoxy)-3-(((2-(dimethylamino)ethoxy)(hydroxy)phosphoryl)oxy)propyl palmitate	Lipids	down
N-(1-Deoxy-1-fructosyl)Piperidic acid	Alkaloids	up
9-Hydroxy-12-oxo-15(Z)-octadecenoic acid	Lipids	down
5-Hydroxycostic acid	Terpenoids	down
Citrinin	Others	down
DGMG 18:3(2n isomer)	Lipids	up
Eriodictyol	Flavonoids	down
2,2'-(3-methylcyclohexane-1,1-diyl)diacetic acid	Organic acids	down
3-Guanidinopropanoate	Organic acids	down
Glucosyl 6,9-dihydroxydec-4-enoic acid	Terpenoids	down
6-Galloyl Isoascorbic acid	Others	down
Hydroperoxylinoleic acid	Lipids	down
Ricinoleic acid methyl ester	Lipids	up
Aflatoxin G1 9,10-epoxide	Others	down
3-Aminosalicylic acid	Phenolic acids	down
Aloe emodin-1-O-glucoside	Quinones	down
Phomolide G	Others	down
Keto-Deoxy-Nonulonic acid	Lipids	down

Terephthalate	Phenolic acids	down
5,7-Dihydroxy-1(3H)-isobenzofuranone	Others	down
2-Hydroxy-4-methyl-3-tridecanoyloxypentanoic acid methyl ester	Lipids	up
N-acetyl-beta-alanine	Amino acids and derivatives	down
2-Oxoglutarate	Organic acids	down
1-O-Vanilloyl-D-Glucose	Phenolic acids	up
5-hydroxy-anthraquinone-2-carboxylic acid	Quinones	up
Cyrtominetin	Flavonoids	down
Glucosyl 5,8-dihydroxy-2,6-dimethylocta-2,6-dienoic acid	Terpenoids	down
3-Hydroxydecanedioic acid	Lipids	up
10-Hydroxy-2-decenoic acid	Lipids	up
Prolylhydroxyproline	Amino acids and derivatives	down
Dihydroartemisinin	Terpenoids	down
10-hydroxymajoroside	Terpenoids	up
Reynosin	Terpenoids	down
Asp-Glu-Tyr	Amino acids and derivatives	down
orthosphenic acid	Terpenoids	down
N-Acetyl-6-hydroxy-L-tryptophan	Amino acids and derivatives	down
2,4,6,4'-Tetrahydroxy-stilbene-2-O-glucoside	Others	down
3-Carboxy-4-Methyl-5-Propyl-2-Furanpropionic Acid	Organic acids	down
Porrigenic acid	Lipids	up
3-Hydroxyurs-12-en-28-oic acid (3-Epiursolic acid)	Terpenoids	down
Urolithin D	Flavonoids	down
Glucosyl 2-Hydroxy-4-Methylpentanoic Acid	Organic acids	down
6-O-Feruloyl-glucose	Phenolic acids	down
Lucyoside F	Terpenoids	down
5-[(1S,2R,3R,5S)-3,5-dihydroxy-2-[(E,3S)-3-hydroxyoct-1-enyl]cyclopentyl]pentanoic acid	Lipids	down
Petasiphenone	Phenolic acids	down
(4-(2-oxopropoxy)phenyl)-L-alanine	Amino acids and derivatives	up
3,23-Dihydroxy-30-noroleana-12,20(29)-dien-28-oic acid (30-Norhederagenin)	Terpenoids	up
3-O-(3-Methoxy-4-hydroxybenzoyl)-4-O-caffeoylquinic acid	Phenolic acids	down
Lactate	Organic acids	down
Oxalate	Organic acids	down
2-Aldehydo-A(1)-norlup-20(29)-en-27,28-dioic acid (Zizyberanal acid)	Terpenoids	up
3-Hydroxyurs-12-en-28-oic acid (Ursolic acid)	Terpenoids	up
3-Hydroxy-9,19-cyclolanost-24-en-26-oic acid (Mangiferolic acid)	Terpenoids	up
16,23:16,30-Diepoxydammar-24-ene-3,20-diol (Jujubogenin)	Terpenoids	down
3,19-Dihydroxyurs-12-en-28-oic acid (Pomolic acid)	Terpenoids	down
3-(3,4-dihydroxyphenyl)propanoic acid glucoside	Phenolic acids	up
(-)-5'-Methoxyisolariciresinol 3alpha-O-beta-glucopyranoside	Lignans and Coumarins	down
Resveratrol-3,5-di-O-glucoside	Others	down

Table S7 Common differential metabolites among the three comparison groups

Compounds	Class I
LysoPE 16:1(2n isomer)	Lipids
LysoPC 17:1	Lipids
2-Ethynylbenzaldehyde	Others
LysoPE 17:1(2n isomer)	Lipids
S-Adenosyl-L-methionine	Amino acids and derivatives
Linolenyl Alcohol	Others
Isocytosine	Nucleotides and derivatives
Epigoitrin	Others
4-carbamoyl-2-(octadeca-9,12-dienamido)butanoic acid	Lipids
Sarmentosin	Alkaloids
3-Hydroxyanthranilate	Alkaloids
7-phenylhept-2-en-1-ol	Others
Ganoderenic Acid A	Terpenoids
Tangeretin	Flavonoids
3',4',5',5,7-Pentamethoxyflavone	Flavonoids
Ganoderic Acid Sigma	Terpenoids
5-Hydroxyquinoline	Alkaloids
3',4',5,5',6,7-Hexamethoxyflavone	Flavonoids
LysoPE 14:0	Lipids
4'-Methoxyacetophenone	Others
PS(18:1/0:0)	Lipids
3α,20β,21β-trihydroxy-16-oxo-serrat-14-en-24-oic acid	Terpenoids
15(R)-Hydroxylinoleic Acid	Lipids
1,6-Dihydroxy-4(14)-eudesmene	Terpenoids
trans-Cinnamate	Phenolic acids
HexosylLPE18:2	Lipids
9-Hydroperoxy-9Z,11E-Octadecadienoic Acid	Lipids
6-Geranylnaringenin; Mimulone; Bonannione A	Flavonoids
Hydroxy ricinoleic acid	Lipids
9,10,11-Trihydroxy-12-octadecenoic acid	Lipids
Phenylpropanoate	Phenolic acids
Phelligridin A	Phenolic acids
DGMG 18:3(2n isomer)	Lipids
Ricinoleic acid methyl ester	Lipids
Terephthalate	Phenolic acids
5,7-Dihydroxy-1(3H)-isobenzofuranone	Others
2-Hydroxy-4-methyl-3-tridecanoyloxypentanoic acid methyl ester	Lipids
10-Hydroxy-2-decenoic acid	Lipids
orthosphenic acid	Terpenoids
5-[(1S,2R,3R,5S)-3,5-dihydroxy-2-[(E,3S)-3-hydroxyoct-1-enyl]cyclopentyl]pentanoic acid	Lipids
